# Springtail phylogeography highlights biosecurity risks of repeated invasions and intraregional transfers among remote islands

**DOI:** 10.1111/eva.12913

**Published:** 2020-02-12

**Authors:** Helena P. Baird, Katherine L. Moon, Charlene Janion‐Scheepers, Steven L. Chown

**Affiliations:** ^1^ School of Biological Sciences Monash University Clayton Victoria Australia; ^2^ Iziko Museums of South Africa Cape Town South Africa; ^3^ Department of Zoology & Entomology University of the Free State Bloemfontein South Africa

**Keywords:** genomics, invasive species, islands, phylogeography, population connectivity, soil biodiversity, springtail

## Abstract

Human‐mediated transport of species outside their natural range is a rapidly growing threat to biodiversity, particularly for island ecosystems that have evolved in isolation. The genetic structure underpinning island populations will largely determine their response to increased transport and thus help to inform biosecurity management. However, this information is severely lacking for some groups, such as the soil fauna. We therefore analysed the phylogeographic structure of an indigenous and an invasive springtail species (Collembola: Poduromorpha), each distributed across multiple remote sub‐Antarctic islands, where human activity is currently intensifying. For both species, we generated a genome‐wide SNP data set and additionally analysed all available COI barcodes. Genetic differentiation in the indigenous springtail *Tullbergia bisetosa* is substantial among (and, to a lesser degree, within) islands, reflecting low dispersal and historic population fragmentation, while COI patterns reveal ancestral signatures of postglacial recolonization. This pronounced geographic structure demonstrates the key role of allopatric divergence in shaping the region's diversity and highlights the vulnerability of indigenous populations to genetic homogenization via human transport. For the invasive species *Hypogastrura viatica*, nuclear genetic structure is much less apparent, particularly for islands linked by regular shipping, while diverged COI haplotypes indicate multiple independent introductions to each island. Thus, human transport has likely facilitated this species’ persistence since its initial colonization, through the ongoing introduction and inter‐island spread of genetic variation. These findings highlight the different evolutionary consequences of human transport for indigenous and invasive soil species. Crucially, both outcomes demonstrate the need for improved intraregional biosecurity among remote island systems, where the policy focus to date has been on external introductions.

## 1 INTRODUCTION

Human transport is eroding the isolation of island ecosystems, with remote islands especially vulnerable to invasion (Moser et al., 2018). Many island biotas now comprise high proportions of introduced species (e.g. Cicconardi et al., 2017; Gaston, Jones, Hänel, & Chown, 2003), which have a range of often substantial impacts (e.g. Clavero, Brotons, Pons, & Sol, 2009; Reaser et al., 2007). Human movements also threaten to merge previously isolated populations of indigenous species, leading to genetic homogenization and subsequent reductions in fitness (Crispo, Moore, Lee‐Yaw, Gray, & Haller, 2011; Olden, 2006), or even reverse speciation (Seehausen, 2006). Ultimately, the response of island communities to increased human transport will be shaped by dispersal, colonization and evolutionary processes unique to both the indigenous and introduced biota (Gillespie, Claridge, & Roderick, 2008). While invasion patterns and dynamics have been well studied for a growing range of island taxa (e.g. Lloret et al., 2005; Moser et al., 2018), those for the soil biota remain poorly known (Coyle et al., 2017; Novo et al., 2015; Ricciardi et al., 2017), despite the prevalence of introduced soil‐dwelling species on islands worldwide (e.g. Cicconardi et al., 2017; Frenot et al., 2005). Changes in soil community composition have far‐reaching effects on ecosystem functioning (e.g. Coyle et al., 2017; Geisen, Wall, & Putten, 2019); therefore, understanding how human transport can alter the structure and evolutionary processes of soil populations is a pressing priority for predicting broader impacts on island systems.

The sub‐Antarctic islands (located in the Southern Ocean) provide an exemplary setting to do so. They are among the world's most remote islands and have substantial conservation value (Chown, Rodrigues, Gremmen, & Gaston, 2001; UNESCO, 2019), with highly specialized soil ecosystems considered vulnerable to invasion (Convey & Lebouvier, 2009; Frenot et al., 2005). Transport across the Southern Ocean is burgeoning as human activities intensify in the Antarctic, increasing the risk of nonindigenous species introductions into the region, as well as intraregional movement of populations between islands or biogeographic zones (Hughes & Convey, 2010; Lee & Chown, 2011). Two key potential consequences have been identified for intraregional transport: the genetic homogenization and biogeographic breakdown of distinct indigenous populations, and the further spread of invasive species established in the region. The genetic structure underlying indigenous and invasive populations will largely determine how this plays out (Crispo et al., 2011; Olden, 2006; Petit, 2004). Thus, an improved understanding of the phylogeography of sub‐Antarctic populations can begin to inform generalities for isolated soil ecosystems under growing transport regimes.

Present understanding of indigenous phylogeographic structure across the terrestrial sub‐Antarctic is based on a limited number of molecular studies (see Moon, Chown, & Fraser, 2017). Instances where few genotypes predominate across the region are typically thought to represent postglacial recolonization of islands following population bottlenecks during glacial maxima (e.g. Fraser, Nikula, Spencer, & Waters, 2009; Wouw, Dijk, & Huiskes, 2008), presumably aided by rafting on marine currents (Fraser, Nikula, Ruzzante, & Waters, 2012; McGaughran, Stevens, Hogg, & Carapelli, 2011). However, many sub‐Antarctic soil populations are now considered genetically isolated with pronounced differentiation between islands (e.g. van Vuuren, Lee, Convey, & Chown, 2018), often indicating allopatric speciation processes (e.g. Allegrucci, Carchini, Convey, & Sbordoni, 2012; Stevens, Greenslade, Hogg, & Sunnucks, 2006). At a finer scale, intra‐island population differentiation has been detected for several species, aligning with historical barriers to gene flow such as glaciers and topography (e.g. Chau et al., 2019; Mortimer, Vuuren, Meiklejohn, & Chown, 2012). Although these studies are limited in geographic and molecular scope (Halanych & Mahon, 2018; Moon et al., 2017), they provide preliminary evidence that the indigenous terrestrial faunas comprise genetically distinct populations vulnerable to homogenization by humans, as is the case for islands elsewhere (e.g. Gillespie et al., 2008; Juan, Crespo, Cowan, Lexer, & Fay, 2004; Vandergast, Gillespie, & Roderick, 2004).

The risk of human activity not only introducing new alien species, but also spreading or reintroducing existing invasives, is also of concern for island systems such as the sub‐Antarctic (Houghton, Terauds, Merritt, Driessen, & Shaw, 2019; Lee & Chown, 2011). Multiple introductions from distinct sources can enhance genetic variation within invasive populations, potentially promoting their establishment and persistence (Dlugosch, Anderson, Braasch, Cang, & Gillette, 2015; Lavergne & Molofsky, 2007; Wagner, Ochocki, Crawford, Compagnoni, & Miller, 2017). The few studies that have employed high‐resolution molecular markers for sub‐Antarctic invasive species have indeed revealed multiple colonizations of a single island (Ouisse, 2016; Piertney et al., 2015), though not always (e.g. Hardouin et al., 2010). The absence of genetic studies on more widespread sub‐Antarctic invasives makes it difficult to determine whether initial introductions have been followed by natural dispersal to other islands, or whether each island represents an additional introduction. Data on the genetic structure of invasive populations are thus essential to better understand invasion dynamics across the region (Estoup & Guillemaud, 2010; Le Roux & Wieczorek, 2009) and to design adequate biosecurity measures for remote island ecosystems (e.g. Abdelkrim, Pascal, Calmet, & Samadi, 2005; Dudaniec, Gardner, Donnellan, & Kleindorfer, 2008).

We therefore conducted the first genome‐wide SNP‐based analysis of phylogeographic structure for an indigenous and an invasive soil species distributed across multiple sub‐Antarctic islands. Given that previous studies in the region primarily examine COI barcodes, we also incorporated COI data to compare mitochondrial outcomes with nuclear genomic patterns. We focussed on Collembola (springtails), which represent a substantial component of sub‐Antarctic soil communities and include a large and concerning number of alien invasive species on these and other islands (Baird, Janion‐Scheepers, Stevens, Leihy, & Chown, 2019; Cicconardi et al., 2017; Greenslade & Convey, 2012).

## 2 MATERIALS AND METHODS

### 2.1 Sampling

**Figure 1:**
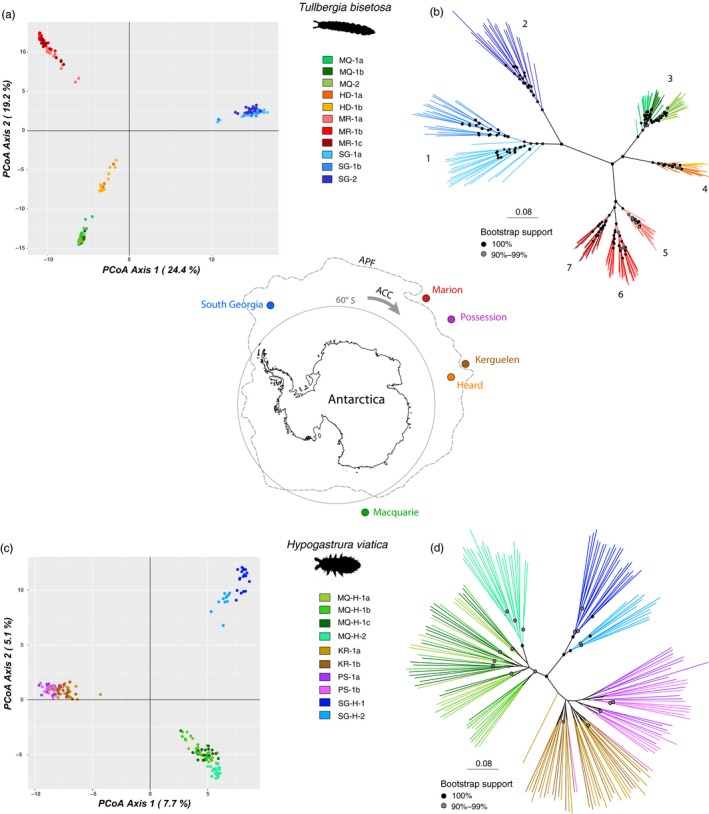
Principal coordinate analysis (PCoA) and maximum likelihood (ML) tree outcomes for the indigenous springtail *Tullbergia bisetosa* (a,b) and the invasive springtail *Hypogastrura viatica* (c,d), based on 5,680 and 7,275 SNPs, respectively. A map of Antarctica showing the sub‐Antarctic islands from which the two species were sampled is depicted in the centre. Individuals in both PCoA plots and ML trees are colour‐coded by their geographic origin; each island has a main colour, and sites within islands are shades of that colour (see legends for each species). Sites within an island discerned by a letter suffix (1a, b, c..) are separated by <5 km; sites within an island discerned by a number suffix (1, 2…) are separated by >10 km. PCoA plots show the per cent variation explained by each axis. Branch lengths on the ML trees represent the average number of substitutions per site (see scale bars), and nodes show bootstrap support (nodes with <90% support are not shown). Numbers on the ML tree for *T. bisetosa* show the groupings inferred by fixed allelic differences to be strongly isolated from gene flow. APF, Antarctic Polar Front; ACC, Antarctic Circumpolar Current

We sampled two springtail species from the Order Poduromorpha: the indigenous *Tullbergia bisetosa* Börner, 1902 (Tullbergiidae), and the highly invasive *Hypogastrura viatica* (Tullberg, 1872) (Hypogastruridae)*.* Both of these species occur on at least four sub‐Antarctic islands and have been confirmed not to comprise cryptic species (*T. bisetosa:* Louis Deharveng, pers. comm.; *H. viatica*: Greenslade & Convey, 2012). To explore small‐ and large‐scale genetic structure*,* multiple sites were sampled from the sub‐Antarctic islands of Macquarie, Heard, Kerguelen, Possession, Marion and South Georgia (see Figure 1) between the years 2004 and 2017 (see Tables S1.1 and S1.2). Given the logistical challenges accessing these islands, the spatial spread of within‐island sampling varied and sample sizes were occasionally limited. Nonetheless, at least two sites were sampled from each island, spaced a minimum of 100 m and a maximum of 21 km apart, while the islands themselves are separated by 1,000–8,000 km of open ocean (see Tables S2.1 and S2.2 for distance matrices). Animals were sampled either by direct aspiration (from an area <30 cm^2^) or by Tullgren funnel extractions of soil or vegetation cores (<30 cm^2^ width), and were immediately preserved in ethanol.

### 2.2 DNA extraction and SNP genotyping

Specimens were sent to Diversity Arrays Technology (DArT) Pty. Ltd. (Canberra, Australia) for genomic DNA extraction and sequencing using DArT‐Seq^TM^ technology. DArT‐Seq^TM^ is a reduced representation next‐generation sequencing method, which employs a combination of restriction enzymes to digest genomic DNA, followed by the ligation of adaptors to both restriction enzyme overhangs, to facilitate sample identification and Illumina sequencing (see methodology in Kilian et al., 2012). Raw Illumina data are processed using a proprietary DArT pipeline to provide a quality‐filtered SNP data set, which we filtered even further using the “dartR” package v.1.0.5 (Gruber, Unmack, Berry, & Georges, 2018) in R v.3.4.3 (R Core Team, 2017). Appendix S1 provides more detail on DArT‐Seq SNP library preparation, quality control and filtering measures.

### 2.3 SNP analyses

For the analyses where statistical significance was required for decision‐making (tests for selection, fixed allelic differences and deviations from the Hardy–Weinberg equilibrium), our results are adjusted for multiple tests using the most common approach in the genomic literature (e.g. Bonferroni, false discovery rate). For all other analyses, we only calculated uncorrected *p*‐values where necessary and generally refrain from heavy emphases on statistical significance, acknowledging growing concern over the appropriate adjustment for multiple testing in genomics (e.g. Sethuraman et al., 2019; White, Ende, & Nichols, 2019) and the over‐interpretation of *p*‐values in general (Amrhein, Greenland, & McShane, 2019).

The final set of SNPs for each species was tested for evidence of selection in BayeScan 2.1 (Foll & Gaggiotti, 2008), using the *F*_ST_ outlier approach and a false discovery rate of *α* = 0.01. Each SNP locus was also tested for deviations from Hardy–Weinberg equilibrium (HWE) in each population, using exact tests with a Bonferroni correction, conducted in dartR.

To examine the genetic diversity within each population, observed and expected heterozygosity and inbreeding coefficients were calculated for each species at each site using GenoDive v.2.0 (Meirmans & Van Tienderen, 2004). The 95% confidence intervals for these indices were generated with 5,000 bootstraps to compare differences in diversity between sites and between islands, within each species.

Population differentiation was initially explored within each species by generating global and pairwise *F*_ST_ estimates (as per Weir & Cockerham, 1984) in GenoDive, using 5,000 permutations to assess significance. Given the hierarchical spacing of our populations, we employed hierarchical AMOVA (Excoffier, Smouse, & Quattro, 1992) to assess the subdivision of genetic variation at each level of sampling (i.e. among islands and among sites within islands). AMOVA was carried out in GenoDive.

Principal coordinate analysis (PCoA) was used to visualize the clustering of genetically similar groups within each species and was carried out using the “gl.pcoa” command in dartR. Additionally, 3D PCAs (which enabled visualization of a third axis) were created using the “pca3d” package (Weiner, 2017) in R. We also used a maximum likelihood approach to infer unrooted phylogenetic trees, as implemented in IQ‐TREE (Nguyen, Schmidt, Haeseler, & Minh, 2014). Node support was inferred based on 10,000 bootstraps of the data, and output files were visualized with FigTree v.1.4.3 (Rambaut, 2009).

Population structure was further explored using the Bayesian clustering algorithm implemented in fastSTRUCTURE (Raj, Stephens, & Pritchard, 2014). Ten replicates were run for each *K* value from *K* = 1 to *K* = 15, using the simple prior model. The number of populations that best fit the data was determined using the “chooseK.py” function, and these population assignments were visualized using Distruct v.2.3 (Chhatre, 2019).

Finally, each data set was tested for fixed allelic differences (when two populations share no alleles at a given SNP), which can indicate a complete lack of contemporary gene flow (Slatkin, 1987). This approach is less affected by departures from HWE and small sample sizes than the more conventional “Structure” family of clustering software (Georges et al., 2018). Fixed difference analysis was conducted in dartR using the “gl.collapse.recursive” function. For each pairwise population comparison, we used 5,000 simulations to estimate the expected rate of false positives (which arise due to the finite number of individuals in each population), and any two populations deemed to have an insignificant number of fixed differences (based on this false‐positive rate, set at *α* = 0.01) were amalgamated. This was performed iteratively until a final set of “pseudo‐populations” remained, each discernable by having a significant number of fixed differences from one another.

### 2.4 Mitochondrial DNA analyses

All available COI sequences for *T. bisetosa* and *H. viatica* were collated from GenBank (http://www.ncbi.nlm.nih.gov/genbank) and the Barcode of Life Data System (“BOLD”: http://www.barcodinglife.org), by searching for public records within their respective Barcode Index Number (BIN) on BOLD. BINs employ a Refined Single Linkage algorithm to cluster sequences that are highly likely to represent the same species (Ratnasingham & Hebert, 2013), and we conservatively excluded any BIN members that had been morphologically identified as a species other than *T. bisetosa* or *H. viatica.* All remaining sequences ≥500 bp within each BIN were downloaded (see Tables S3.1 and S3.2 for accession numbers), manually checked for stop codons or indels that could indicate pseudogenes, and aligned using MUSCLE in MEGA v.7 (Kumar, Stecher, & Tamura, 2016). Haplotype networks were created using the median‐joining option (Bandelt, Forster, & Röhl, 1999) in PopART (popart.otago.ac.nz). Further exploration of the data, such as testing for population expansion, was not attempted given the opportunistic (and therefore geographically nonuniform) sampling of the sequences.

## 3 RESULTS

### 3.1 SNP data sets

The DArTsoft14 pipeline delivered an initial 39,847 and 37,803 SNPs for *Tullbergia bisetosa* and *Hypogastrura viatica*, respectively. After quality filtering (see Table S4 for the data set size at each filtering step), the final data set for *T. bisetosa* comprised 5,680 SNPs (see Baird, Moon, Janion‐Scheepers, & Chown, 2019a), and for *H. viatica* comprised 7,275 SNPs (see Baird, Moon, Janion‐Scheepers, & Chown, 2019b). Less than 5% of SNPs were found to significantly violate HWE in any single population for either species, and the vast majority of these HWE deviations represented heterozygote deficits (>95% for both species). The number of outlier SNPs putatively under selection was also minimal, representing 2% of the data set for both species (123 SNPs for *T. bisetosa* and 158 SNPs for *H. viatica*). Thus, outlier SNPs putatively under selection or deviating from HWE were not removed from the main data sets. However, we prepared a conservative data set for each species with all of these SNPs removed (see Table S4) on which we performed a subset of the analyses (genetic diversity statistics, AMOVA and PCoA), to verify that this did not affect our results. These outcomes are almost identical to those from the main data sets (see Figures S1, S2 and Tables S5–S8); we henceforth refer only to results from the full data sets.

### 3.2 Genomic diversity and structure

#### 3.2.1 *Tullbergia bisetosa*

Populations of *T. bisetosa* had low levels of observed and expected heterozygosity (global *H*_O_ = 0.069, global *H*_E_ **= 0.099; see Table 1 for local population values), in part reflecting the large proportion of SNPs that were fixed for a single allele within each population. Heterozygote deficiencies were evident for all populations by high and positive coefficients of inbreeding (global *F*_IS_ = 0.304; Table 1), indicating that nearly one third of heterozygosity was lost. Comparisons between populations of *T. bisetosa* revealed that South Georgia harboured significantly higher levels of genetic diversity (based on observed and expected heterozygosity) than Macquarie, Heard or Marion islands (*p* < .05 for both *H*_O_ and *H*_E_; Table 1).

**Table 1:** Sample size and genetic diversity statistics (with 95% CIs in parentheses) for sub‐Antarctic populations of the indigenous springtail *Tullbergia bisetosa* and the invasive springtail *Hypogastrura viatica,* based on 5,680 and 7,275 genome‐wide SNPs, respectively

	Site	*N*	*H*_O_	*H*_E_	*F*_IS_
*T. bisetosa*					
Macquarie	MQ‐1a	17	0.046 (0.043, 0.049)	0.067 (0.063, 0.071)	0.316 (0.295, 0.336)
	MQ‐1b	10	0.051 (0.047, 0.054)	0.069 (0.065, 0.073)	0.263 (0.238, 0.288)
	MQ‐2	19	0.059 (0.055, 0.062)	0.069 (0.066, 0.073)	0.156 (0.137, 0.175)
Heard	HD‐1a	4	0.058 (0.052, 0.059)	0.075 (0.067, 0.076)	0.225 (0.185, 0.264)
	HD‐1b	13	0.052 (0.048, 0.054)	0.069 (0.064, 0.072)	0.250 (0.223, 0.278)
Marion	MR‐1a	16	0.046 (0.043, 0.049)	0.071 (0.067, 0.075)	0.353 (0.332, 0.375)
	MR‐1b	23	0.069 (0.065, 0.072)	0.103 (0.098, 0.107)	0.333 (0.316, 0.350)
	MR‐1c	17	0.056 (0.053, 0.060)	0.093 (0.088, 0.097)	0.392 (0.373, 0.410)
Sth Georgia	SG‐1a	20	**0.106 (0.102, 0.110)**	**0.159 (0.154, 0.164)**	0.332 (0.318, 0.346)
	SG‐1b	19	**0.111 (0.107, 0.115)**	**0.153 (0.148, 0.158)**	0.274 (0.259, 0.289)
	SG‐2	20	**0.110 (0.105, 0.113)**	**0.159 (0.153, 0.164)**	0.312 (0.297, 0.327)
	*Global*	178	0.069 (0.068, 0.071)	0.099 (0.098, 0.101)	0.304 (0.296, 0.312)
*H. viatica*					
Macquarie	MQ‐H‐1a	16	0.138 (0.135, 0.142)	0.231 (0.226, 0.235)	0.399 (0.389, 0.410)
	MQ‐H‐1b	22	0.151 (0.148, 0.154)	0.229 (0.225, 0.233)	0.341 (0.332, 0.350)
	MQ‐H‐1c	14	0.133 (0.129, 0.136)	0.229 (0.225, 0.234)	0.421 (0.411, 0.431)
	MQ‐H‐2	21	0.144 (0.141, 0.148)	0.217 (0.213, 0.221)	0.334 (0.325, 0.344)
Kerguelen	KR‐1a	19	0.150 (0.147, 0.154)	0.231 (0.227, 0.236)	0.351 (0.342, 0.360)
	KR‐1b	19	0.143 (0.139, 0.146)	0.229 (0.225, 0.234)	0.378 (0.368, 0.387)
Possession	PS‐1a	17	0.147 (0.143, 0.150)	0.223 (0.218, 0.227)	0.341 (0.330, 0.351)
	PS‐1b	19	0.148 (0.144, 0.151)	0.227 (0.222, 0.231)	0.348 (0.339, 0.358)
Sth Georgia	SG‐H‐1	20	0.133 (0.129, 0.136)	0.218 (0.213, 0.223)	0.390 (0.380, 0.400)
	SG‐H‐2	11	0.111 0.107, 0.114)	0.221 (0.216, 0.225)	0.498 (0.486, 0.509)
	*Global*	178	0.140 (0.137, 0.142)	0.225 (0.222, 0.228)	0.380 (0.374, 0.385)

Note

Sites within an island discerned by a letter suffix (1a, b, c..) are separated by <5 km; sites within an island discerned by a number suffix (1, 2…) are separated by >10 km. Diversity indices for *T. bisetosa* from South Georgia were found to significantly differ from all other islands (*p* < .05), as shown in bold.

Abbreviations: *H*_O_, observed heterozygosity; *H*_E_, expected heterozygosity; *F*_IS_, inbreeding coefficient; *N*, sample size.

Substantial genetic differentiation was found across all populations for *T. bisetosa* (global *F*_ST_ = 0.542), with *F*_ST_ estimates as high as 0.677 between sites on different islands and estimates ranging from 0.027 to 0.346 among sites within an island (Table S9). The largest degree of variation occurred between islands (40%), with a small amount partitioned between sites within islands (10%) and the remaining variance explained by among‐ and within‐individual variation (hierarchical AMOVA; Table 2). The PCoA for *T. bisetosa* also demonstrated a high degree of clustering by island, which captured 43.6% of variation within the first two axes, with subtle clustering by site also evident within South Georgia and Marion Island (Figure 1a). Genetic variation captured by a third PC axis dropped to 5.5%, with this axis further exaggerating the differences between island and, to some degree, the clustering of sites within South Georgia and Marion Island (Figure S3).

**Table 2:** Analysis of molecular variance (AMOVA) outcomes for *Tullbergia bisetosa* and *Hypogastrura viatica* based on 5,680 and 7,275 genome‐wide SNPs, respectively

	*T. bisetosa*			*H. viatica*		
	SS	% var	*F*‐statistic	SS	% var	*F*‐statistic
Among island	82,165.13	40.0	0.40	29,815.00	10.1	0.10
Among site within island	19,914.76	10.3	0.17	10,565.68	1.9	0.02
Among samples within site	77,553.09	14.4	0.29	186,693.77	29.0	0.33
Within samples	45,497.50	35.3	0.65	99,756.50	59.0	0.41

Abbreviations: % var, per cent of total variation; SS, sum of squares.

The maximum likelihood (ML) tree for *T. bisetosa* was well supported with most nodes having >90% bootstrap support (Figure 1b). Each island formed a distinct clade, and further clear groupings were associated with intra‐island sites for South Georgia and Marion Island (these islands also had the highest *F*_ST_ values between their sites; Table S9). Despite having some of the furthest spread intra‐island sites, Macquarie Island only showed weak grouping by site and intra‐island clades were not discernible within Heard Island.

Seven clusters (*K* = 7) were identified by fastSTRUCTURE to best represent the data for *T. bisetosa*, although the resultant structure plot showed five clusters to be most explicit (Figure S4). These corresponded to each island, with an extra division separating the furthest‐spaced sites on South Georgia, as was clear on the ML tree also. An additional cluster was assigned to individuals at low frequency and sporadically across all source populations, which may represent admixture with an unsampled island (*T. bisetosa* is also found on Prince Edward Island and the South Sandwich Islands, which we were unable to sample), or missing data occurring at the same SNPs.

Numerous fixed allelic differences were evident between populations of *T. bisetosa* (Table S10). The amalgamation of populations without significant fixed differences resulted in seven discrete “pseudo‐populations” separated by an inferred lack of gene flow. These are indicated by numbers 1–7 on the ML Tree (Figure 1b) and correspond closely to the clustering of branches within the tree, representing the four islands plus additional subdivisions within South Georgia and Marion Island. These pseudo‐populations were also largely in keeping with the fastSTRUCTURE clusters.

#### 3.2.2 *Hypogastrura viatica*

Diversity estimates for each population of *H. viatica* are provided in Table 1; none of the populations were found to differ significantly from one another in observed or expected heterozygosity (global *H*_O_ = 0.140, global *H*_E_ **= 0.225). Populations consistently exhibited high levels of inbreeding, ranging from *F*_IS_ = 0.334 to *F*_IS_ = 0.498 (global *F*_IS_ = 0.380; Table 1) indicating that up to half of the expected heterozygosity was lost.

Genetic differentiation across all populations of *H. viatica* was modest (global *F*_ST_ = 0.115; Table S11). The vast majority of genetic variation occurred within sites or individuals (88% total), with a small amount partitioned between islands (10%) and a negligible amount between sites within islands (2%) (hierarchical AMOVA; Table 2). Weak geographic structure was also demonstrated by the PCoA, which, although revealing a clustering by island (with the exception of the Possession and Kerguelen that clustered together), only captured 12.8% of variation within the first two axes (Figure 1c). Visualization of a third axis revealed further differentiation between sites on South Georgia and Macquarie Island, though only explaining an additional 1.3% of variation (Figure S5).

The ML tree for *H. viatica* was weakly supported at several nodes, and, in line with AMOVA results, branch lengths between individuals within populations were longer than branches between islands (Figure 1d). Three clades were evident, however, corresponding with the three clusters in the PCoA (South Georgia, Macquarie Island and Possession + Kerguelen Islands) and weak intra‐island clades for South Georgia and Macquarie Island. Three clusters (*K* = 3) were also identified by fastSTRUCTURE (Figure S6), which were in agreement with the main island groupings in the PCoA and ML tree, and which again did not discriminate between Possession and Kerguelen Islands.

There were very few fixed allelic differences between populations of *H. viatica* (Table S12), and none were above the false‐positive rate. Thus, none of the populations could be distinguished by a complete lack of gene flow; rather, they were all amalgamated into a single pseudo‐population.

### 3.3 Mitochondrial DNA diversity

#### 3.3.1 *Tullbergia bisetosa*

**Figure 2:**
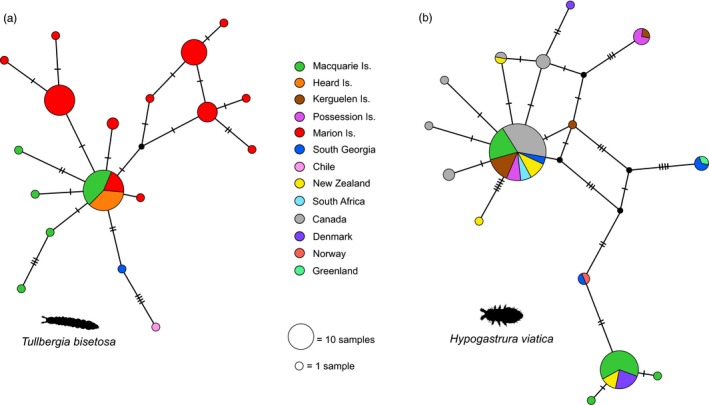
Median‐joining haplotype networks for the indigenous springtail *Tullbergia bisetosa* (a) and the invasive springtail *Hypogastrura viatica* (b), based on COI sequences retrieved from GenBank and BOLD. Circle size is proportional to the haplotype frequency, black nodes represent inferred unsampled haplotypes, and hatches show the number of inferred mutations between haplotypes. Sequences are colour‐coded by their country or island of origin

A total of 70 COI sequences were retrieved for *T. bisetosa,* originating from South Georgia, Marion, Macquarie and Heard Islands, as well as a single sequence from Chile (Table S3.1). The haplotype network is provided in Figure 2a; most haplotypes from the sub‐Antarctic islands were separated by one or two mutational steps. The nine sequences from Heard Island represented a single haplotype, which was also the most frequent haplotype at Macquarie Island and additionally present on Marion Island. Limited observations can be made for South Georgia, which was only represented by a single sequence, although this haplotype was the closest related to the only continental haplotype (from Chile).

#### 3.3.2 *Hypogastrura viatica*

For *H. viatica*, 94 COI sequences were retrieved originating from South Georgia, Macquarie, Possession and Kerguelen Islands, plus an additional six countries globally (Table S3.2); the haplotype network is shown in Figure 2b. The most common haplotype had a global distribution, occurring on all four sub‐Antarctic islands and Canada, New Zealand and South Africa, and often at relatively high frequency. Additional haplotypes from the sub‐Antarctic islands were separated from this common global haplotype (and from each other) by several mutational steps. Possession and Kerguelen Islands shared a haplotype, which was at least five mutations from the common haplotype. Even further diverged (separated from the common haplotype by at least six mutations) were two distinct haplotypes from South Georgia—one shared with Greenland, the other with Norway—and a haplotype occurring at relatively high frequency on Macquarie Island, which was shared with New Zealand and Denmark.

## 4 DISCUSSION

We present some of the first nuclear genomic data for sub‐Antarctic soil fauna, revealing that gene flow between sub‐Antarctic islands is highly restricted in the indigenous springtail *Tullbergia bisetosa*, with structure also evident within some islands. Numerous COI haplotypes are shared among islands, however, potentially reflecting postglacial colonization. For the invasive springtail *Hypogastrura viatica*, genomic structure is much less apparent, particularly for islands linked by human transport, though broad island groupings remain discernible and diverged COI haplotypes reflect a diverse invasion history. Bearing in mind that each species was represented by a unique SNP data set, our findings suggest that increased human movement throughout the sub‐Antarctic is likely to have very different evolutionary consequences for indigenous and invasive soil communities, with wider implications for inter‐island biosecurity policy and for island systems globally.

### 4.1 Pronounced phylogeographic structure in *Tullbergia bisetosa*

Within *T. bisetosa* populations, genetic diversity was low and substantial heterozygote deficits indicated inbreeding. While null alleles potentially contributed, they are unlikely to generate such high inbreeding coefficients alone, particularly given the SNPs were filtered by read depth and call rate (Andrews, Good, Miller, Luikart, & Hohenlohe, 2016). Inbreeding may have been partly due to assortative mating or facultative parthenogenesis, which have been observed in some springtail species (Chernova, Potapov, Savenkova, & Bokova, 2010; Van der Wurff, Isaaks, Ernsting, & Straalen, 2003). Indeed, nonrandom mating would also contribute to the high levels of genetic isolation between populations and, together with the large degree of fixation, may help explain the low overall diversity (global *H*_E_ = 0.099) of *T. bisetosa* populations (Charlesworth, 2003).

Populations of *T. bisetosa* from different sub‐Antarctic islands share little to no gene flow, as revealed by all methods of genomic analysis. The large number of SNPs fixed for different alleles suggests that island populations are likely on independent evolutionary trajectories and allopatric speciation between islands may already be underway. Indeed, a large proportion of springtails from the Southern Ocean region are endemic to a single island (Baird, Janion‐Scheepers, et al., 2019), a situation likely promoted by allopatric genetic divergence (Losos & Ricklefs, 2009). Lacking flight, springtails disperse passively; therefore, genetic isolation over thousands of kilometres of open ocean is not surprising (see Costa et al., 2013).

Populations of *T. bisetosa* separated by several kilometres within both Marion Island and South Georgia were also strongly differentiated, as indicated by high *F*_ST_ values, fixed allelic differences and distinct clades on the ML tree. In contrast, populations within Heard Island and Macquarie Island were not discernible by ML nor by fixed difference analysis, with much lower *F*_ST_ values. While this may reflect the smaller geographic distance separating Heard Island populations (<1 km), populations on Macquarie Island were separated by up to 21 km, vastly exceeding the distances between differentiated Marion Island populations (1–3 km). Differences in local population structure of *T. bisetosa* may therefore reflect the unique geological history of each island. South Georgia and Marion Island have undergone numerous pulses of glaciation (and volcanism on the latter), which would have promoted population differentiation over time through physical fragmentation (e.g. Mortimer et al., 2012; Myburgh, Chown, Daniels, & Vuuren, 2007), potentially also explaining the higher genetic diversity on South Georgia. In contrast, Macquarie Island has not experienced glacial or volcanic activity, while Heard Island has been so extensively glaciated that it is unlikely to have been colonized by *T. bisetosa* until relatively recently, following deglaciation (Hodgson et al., 2014). Signatures of this postglacial colonization may also help to explain the seemingly incongruent mitochondrial results observed for *T. bisetosa*.

The COI data set for *T. bisetosa* revealed very different patterns to the nuclear genomic data, demonstrating the tenuousness of COI‐based inferences for sub‐Antarctic fauna to date (also see Halanych & Mahon, 2018). The sharing of a common COI haplotype between Marion, Heard and Macquarie Islands could be inferred to show a high degree of connectedness between populations; considering SNP data however, it is clear that there is presently negligible nuclear gene flow between islands. Rather, this haplotype may reflect the retention of an ancient polymorphism involved in the initial colonization of these islands (Fraser et al., 2012), particularly given its central relationship to most other variants. That it was the only haplotype found on Heard Island (from nine samples covering multiple sites) is indicative of founder effect, in accordance with a more recent, postglacial colonization of this island (see Wouw et al., 2008, for similar observations in plants). Alternatively, it remains possible that the lack of any clear mitochondrial subdivision between islands reflects selection acting on COI or another region of the mitochondrial genome (Ballard & Whitlock, 2004; Dowling, Friberg, & Lindell, 2008).

Our findings for *T. bisetosa* substantiate concerns that human transport of biota between sub‐Antarctic islands may lead to genetic homogenization of otherwise highly structured species (Hughes & Convey, 2010; Lee & Chown, 2011). The low genetic diversity, high inbreeding coefficients and marked genetic differentiation observed for *T. bisetosa* may indicate localized adaptation of populations, which could be swamped by the transport of individuals between islands, resulting in decreased fitness (“migration load”: see Bolnick & Nosil, 2007; Lenormand, 2002). Breaching the isolation between islands with human‐mediated gene flow could even disrupt the progress of allopatric speciation and associated biodiversity outcomes (Crispo et al., 2011; Seehausen, 2006). If *T. bisetosa* populations are, however, reproductively isolated and can no longer introgress, inter‐island exchange of these newly speciated populations remains a threat to the region's biogeography, which boasts a high degree of endemicity (Baird, Janion‐Scheepers, et al., 2019). The ultimate course of these microevolutionary processes depends largely on the degree of selection acting on populations (Bourne et al., 2014; Rieseberg & Burke, 2001) and may play out differently for nuclear versus mitochondrial genomes (see Toews & Brelsford, 2012). Regardless, allopatric population divergence plays a significant role in generating island biodiversity (Losos & Ricklefs, 2009); thus, any disruptions to this process via transport‐driven homogenization could pose a threat to island diversity patterns globally. Whether this is especially the case for soil fauna requires testing across other taxa, though high population structure is indeed an emerging characteristic of indigenous soil biota elsewhere (e.g. Costa et al., 2013; von Saltzwedel, Scheu, & Schaefer, 2016).

### 4.2 Human‐mediated gene flow and multiple invasions in *Hypogastrura viatica*

Most of the genomic variation within *H. viatica* was partitioned within rather than among populations, with strong levels of inbreeding indicated by heterozygote deficits. High inbreeding coefficients in *H. viatica* may reflect similar mechanisms to those discussed for *T. bisetosa*; in particular, parthenogenesis has previously been proposed to underlie the successful invasion history of *H. viatica* (Greenslade, 2002). Genetic diversity was not particularly low within *H. viatica* populations (global *H*_E_ = 0.225), as would be expected if populations had been seeded by a single colonization of few individuals. Indeed, COI data indicated that multiple introductions have occurred on each island, a process known to maintain genetic diversity in other invasive populations (Kolbe, Larson, Losos, & Queiroz, 2008; Lavergne & Molofsky, 2007; Garnas et al., 2016). All of the islands contained representatives of a common global COI haplotype, as well as additional unique haplotypes shared with individuals from different countries. Most of the haplotypes within each island were diverged by numerous mutations and thus are much more likely to reflect colonization by differentiated *H. viatica* lineages than in situ diversification (see Piertney et al., 2015). The mixing of lineages from different colonization events helps to explain the large proportion of genomic variation attributed within populations of *H. viatica* rather than at higher geographic levels, and may also be contributing to the heterozygote deficits observed (via Wahlund effect) if different lineages within islands are yet to completely interbreed (see Kolbe et al., 2008).

Although nuclear genomic structure was not particularly pronounced for *H. viatica*, PCoA and fastSTRUCTURE plots did discern clearly between three main island groups, with only Kerguelen and Possession island populations relatively indistinguishable. Population structure was also weakly evident between the two sites on South Georgia and the most distal sites on Macquarie Island, revealing that some degree of genetic drift has occurred within and among islands, since the introduction of *H. viatica*. That this geographic differentiation remained only moderate, however, explaining such a small amount of total genetic variation, likely reflects the limited evolutionary time over which the species has existed in the region, meaning it is unlikely to be at migration–drift equilibrium (Slatkin, 1987). Despite being separated by >1,000 km of open ocean, populations from the islands of Kerguelen and Possession were profoundly more homogenous than populations merely 15 km apart within other islands. Kerguelen and Possession are both governed by France and therefore receive cargo from the same ship annually (Frenot, Gloaguen, Massé, & Lebouvier, 2001), a link that no other islands in our study share. This indicates that shipping has played a role in facilitating genetic exchange of the invasive populations or at least that the two islands have been colonized from the same source population, demonstrating the greater influence of human transport over natural dispersal in structuring *H. viatica* across the sub‐Antarctic. Very similar findings have also been made for invasive aphids from Kerguelen and Possession Islands (Lebouvier et al., 2011).

One COI haplotype for *H. viatica* occurred on all four sub‐Antarctic islands and most other sampled countries, indicating potentially rapid global spread of a successful genotype (Le Roux, Wieczorek, Wright, & Tran, 2007; Garnas et al., 2016). Yet, the haplotypes unique to each island provided most insight on the colonization history for *H. viatica*. The occurrence of two haplotypes on South Georgia shared with Norway and Greenland directly accords with Norway's whaling history on the two islands, which saw substantial shipping transport between them (Tønnessen & Johnsen, 1982). Similarly, the sharing of a Macquarie Island haplotype with New Zealand, the most proximal land mass with historic, science and tourism links to the island, is unsurprising. A connection with Denmark is also in keeping with the long history of Danish vessels chartered to access Macquarie Island (Kriwoken & Williamson, 1993). Finally, the sharing of a unique haplotype solely between Kerguelen and Possession Islands likely reflects their mutual visitation exclusively by France, as for the SNP data. Thus, the unique nature of the sub‐Antarctic islands, each governed and visited by different nations, may influence the population structure of introduced species in the region, with discrete colonizations from different sources more evident than range expansion from a single introduction (also see Baird, Janion‐Scheepers, et al., 2019). It is, however, important to note that *H. viatica* occurs on several more islands across the Southern Ocean than were sampled in this study, for which the colonization history may well differ.

In the context of increased human activity throughout the sub‐Antarctic, the relatively weak geographic structure and negligible fixed allelic differences for *H. viatica* imply that movement of these individuals between islands is unlikely to disrupt evolutionary trajectories or biogeographic patterns to the same degree as for *T. bisetosa*. Rather, intraregional transport may help to spread beneficial alleles introduced by the unique colonizations of each island, an important prerequisite to realizing any advantage from the diversity provided by multiple introductions (Barker et al., 2019; Dlugosch & Parker, 2008). The connectivity among islands linked by shipping indicates that ongoing inter‐island transport has the potential to spread *H. viatica* beyond what might be achieved through natural range expansion. This is likely to apply to other invasive soil species, as natural dispersal is often limited belowground (Cicconardi et al., 2017; Costa et al., 2013). For islands already invaded by *H. viatica*, additional introductions may continue to contribute new genetic variation, as evidenced by differentiated COI lineages. While genetic diversity is not always a prerequisite for invasion success (see Dlugosch et al., 2015), it is likely to enhance an invasive's evolutionary persistence and spread (e.g. Ahlroth, Alatalo, Holopainen, Kumpulainen, & Suhonen, 2003; Lavergne & Molofsky, 2007; Wagner et al., 2017). Therefore, the management concern of multiple introductions (see Le Roux & Wieczorek, 2009) now also appears pertinent to remote island soil systems (see also Novo et al., 2015).

### 4.3 Biosecurity implications

Soil is a key pathway by which humans unknowingly transport species beyond their natural range (Hulme, 2015); therefore, insights from soil biota are particularly relevant to biosecurity. Our study provides evidence that indigenous sub‐Antarctic soil fauna may be vulnerable to genetic homogenization by inter‐island transport, with potential consequences for population fitness, biodiversity processes and/or biogeography. While it is evident, at least for our focal species, that such transport is yet to occur, the degree and extent of biotic exchange will likely increase in the future due to expanding tourism and science activities involving multiple islands (e.g. Stewart, Espiner, Liggett, & Taylor, 2017; Walton & Thomas, 2018). The threat of inter‐island homogenization likely applies to remote islands globally—while generally understudied, other island‐based soil faunas also show evidence of naturally high genetic and biogeographic structure (e.g. Caruso, Diega, & Bernini, 2005; Cicconardi, Nardi, Emerson, Frati, & Fanciulli, 2010). Substantial allopatric genetic drift between populations, as demonstrated here, is known to play a key role in driving island diversity worldwide (Losos & Ricklefs, 2009). Therefore, our findings emphasize calls to consider the genetic consequences of human transport, in order to conserve biodiversity processes (Hudson, Viard, Roby, & Rius, 2016; Petit, 2004; Seehausen, 2006).

Our work also indicates that repeat introductions of existing invasives are an important management consideration for remote island systems, with invasive spread likely promoted by intraregional transport. Again, this is in line with recent findings for soil ecosystems elsewhere (e.g. Novo et al., 2015; Porco et al., 2013). While most studies concerning island biosecurity risks have focused on novel, external introductions, inter‐island transfers are now being recognized as a major concern globally (e.g. IUCN, 2018; Lee & Chown, 2011; Petit, Hoddle, Grandgirard, Roderick, & Davies, 2009). For the sub‐Antarctic islands, which are largely World Heritage‐listed, stringent among‐island biosecurity measures should be considered and would serve as an exemplar for conserved island systems elsewhere.

## CONFLICT OF INTEREST

None declared.

## Data Availability

The SNP data sets for each species have been made publicly available on Figshare at https://doi.org/10.26180/5d6e10736bed4 (*Tullbergia bisetosa*) and https://doi.org/10.26180/5d6f35ed1e4f7 (*Hypogastrura viatica*). Accession numbers for COI sequences are provided in the Appendix S1, with all sequences publicly available on GenBank or BOLD.

## References

[eva12913-bib-0001] Abdelkrim, J. , Pascal, M. , Calmet, C. , & Samadi, S. (2005). Importance of assessing population genetic structure before eradication of invasive species: Examples from insular Norway rat populations. Conservation Biology, 19, 1509–1518. 10.1111/j.1523-1739.2005.00206.x

[eva12913-bib-0002] Ahlroth, P. , Alatalo, R. V. , Holopainen, A. , Kumpulainen, T. , & Suhonen, J. (2003). Founder population size and number of source populations enhance colonization success in waterstriders. Oecologia, 137, 617–620. 10.1007/s00442-003-1344-y 14534781

[eva12913-bib-0003] Allegrucci, G. , Carchini, G. , Convey, P. , & Sbordoni, V. (2012). Evolutionary geographic relationships among orthocladine chironomid midges from maritime Antarctic and sub‐Antarctic islands. Biological Journal of the Linnean Society, 106, 258–274. 10.1111/j.1095-8312.2012.01864.x

[eva12913-bib-0004] Amrhein, V. , Greenland, S. , & McShane, B. (2019). Scientists rise up against statistical significance. Nature, 567, 305–307. 10.1038/d41586-019-00857-9 30894741

[eva12913-bib-0005] Andrews, K. R. , Good, J. M. , Miller, M. R. , Luikart, G. , & Hohenlohe, P. A. (2016). Harnessing the power of RADseq for ecological and evolutionary genomics. Nature Reviews Genetics, 17, 81–92. 10.1038/nrg.2015.28 PMC4823021 26729255

[eva12913-bib-0006] Baird, H. P. , Janion‐Scheepers, C. , Stevens, M. I. , Leihy, R. I. , & Chown, S. L. (2019). The ecological biogeography of indigenous and introduced Antarctic springtails. Journal of Biogeography, 46, 1959–1973. 10.1111/jbi.13639

[eva12913-bib-0007] Baird, H. P. , Moon, K. L. , Janion‐Scheepers, C. , & Chown, S. L. (2019a). SNP dataset for the springtail *Tullbergia bisetosa* Figshare, 10.26180/5d6e10736bed4

[eva12913-bib-0008] Baird, H. P. , Moon, K. L. , Janion‐Scheepers, C. , & Chown, S. L. (2019b). SNP dataset for the springtail *Hypogastrura viatica* Figshare, 10.26180/5d6f35ed1e4f7

[eva12913-bib-0009] Ballard, J. W. O. , & Whitlock, M. C. (2004). The incomplete natural history of mitochondria. Molecular Ecology, 13, 729–744. 10.1046/j.1365-294X.2003.02063.x 15012752

[eva12913-bib-0010] Bandelt, H.‐J. , Forster, P. , & Röhl, A. (1999). Median‐joining networks for inferring intraspecific phylogenies. Molecular Biology and Evolution, 16, 37–48. 10.1093/oxfordjournals.molbev.a026036 10331250

[eva12913-bib-0011] Barker, B. S. , Cocio, J. E. , Anderson, S. R. , Braasch, J. E. , Cang, F. A. , Gillette, H. D. , & Dlugosch, K. M. (2019). Potential limits to the benefits of admixture during biological invasion. Molecular Ecology, 28, 100–113. 10.1111/mec.14958 30485593 PMC6344275

[eva12913-bib-0012] Bolnick, D. I. , & Nosil, P. (2007). Natural selection in populations subject to a migration load. Evolution, 61, 2229–2243. 10.1111/j.1558-5646.2007.00179.x 17767592

[eva12913-bib-0013] Bourne, E. C. , Bocedi, G. , Travis Justin, M. J. , Pakeman Robin, J. , Brooker Rob, W. , & Schiffers, K. (2014). Between migration load and evolutionary rescue: Dispersal, adaptation and the response of spatially structured populations to environmental change. Proceedings of the Royal Society B: Biological Sciences, 281, 20132795. 10.1098/rspb.2013.2795 PMC3906938 24452022

[eva12913-bib-0014] Caruso, T. , La Diega, R. N. , & Bernini, F. (2005). The effects of spatial scale on the assessment of soil fauna diversity: Data from the oribatid mite community of the Pelagian Islands (Sicilian Channel, southern Mediterranean). Acta Oecologica, 28, 23–31. 10.1016/j.actao.2005.01.006

[eva12913-bib-0015] Charlesworth, D. (2003). Effects of inbreeding on the genetic diversity of populations. Philosophical Transactions of the Royal Society of London. Series B: Biological Sciences, 358, 1051–1070. 12831472 10.1098/rstb.2003.1296 PMC1693193

[eva12913-bib-0016] Chau, J. H. , Born, C. , McGeoch, M. A. , Bergstrom, D. , Shaw, J. , Terauds, A. , … van Vuuren, B. J. (2019). The influence of landscape, climate, and history on spatial genetic patterns in keystone plants (*Azorella*) on sub‐Antarctic islands. Molecular Ecology, 28, 3291–3305. 31179588 10.1111/mec.15147

[eva12913-bib-0017] Chernova, N. M. , Potapov, M. B. , Savenkova, Y. Y. , & Bokova, A. I. (2010). Ecological significance of parthenogenesis in collembola. Entomological Review, 90, 23–38. 10.1134/S0013873810010033

[eva12913-bib-0018] Chhatre, V. E. (2019). Distruct v2.3, A modified cluster membership plotting script. Retrieved from http://distruct2.popgen.org

[eva12913-bib-0019] Chown, S. L. , Rodrigues, A. S. L. , Gremmen, N. J. M. , & Gaston, K. (2001). World Heritage status and conservation of southern ocean islands. Conservation Biology, 15, 550–557. 10.1046/j.1523-1739.2001.015003550.x

[eva12913-bib-0020] Cicconardi, F. , Borges, P. A. V. , Strasberg, D. , Oromí, P. , López, H. , Pérez‐Delgado, A. J. , … Emerson, B. C. (2017). MtDNA metagenomics reveals large‐scale invasion of belowground arthropod communities by introduced species. Molecular Ecology, 26, 3104–3115. 10.1111/mec.14037 28139037

[eva12913-bib-0021] Cicconardi, F. , Nardi, F. , Emerson, B. C. , Frati, F. , & Fanciulli, P. P. (2010). Deep phylogeographic divisions and long‐term persistence of forest invertebrates (Hexapoda: Collembola) in the North‐Western Mediterranean basin. Molecular Ecology, 19, 386–400. 10.1111/j.1365-294X.2009.04457.x 20015142

[eva12913-bib-0022] Clavero, M. , Brotons, L. , Pons, P. , & Sol, D. (2009). Prominent role of invasive species in avian biodiversity loss. Biological Conservation, 142, 2043–2049. 10.1016/j.biocon.2009.03.034

[eva12913-bib-0023] Convey, P. , & Lebouvier, M. (2009). Environmental change and human impacts on terrestrial ecosystems of the sub‐Antarctic islands between their discovery and the mid‐twentieth century. Papers and Proceedings of the Royal Society of Tasmania, 143(1), 33–44. 10.26749/rstpp.143.1.33

[eva12913-bib-0024] Costa, D. , Timmermans, M. J. T. N. , Sousa, J. P. , Ribeiro, R. , Roelofs, D. , & Van Straalen, N. M. (2013). Genetic structure of soil invertebrate populations: Collembolans, earthworms and isopods. Applied Soil Ecology, 68, 61–66. 10.1016/j.apsoil.2013.03.003

[eva12913-bib-0025] Coyle, D. R. , Nagendra, U. J. , Taylor, M. K. , Campbell, J. H. , Cunard, C. E. , Joslin, A. H. , … Callaham, M. A. (2017). Soil fauna responses to natural disturbances, invasive species, and global climate change: Current state of the science and a call to action. Soil Biology and Biochemistry, 110, 116–133. 10.1016/j.soilbio.2017.03.008

[eva12913-bib-0026] Crispo, E. , Moore, J.‐S. , Lee‐Yaw, J. A. , Gray, S. , & Haller, B. (2011). Broken barriers: Human‐induced changes to gene flow and introgression in animals: An examination of the ways in which humans increase genetic exchange among populations and species and the consequences for biodiversity. BioEssays, 33, 508–518. 10.1002/bies.201000154 21523794

[eva12913-bib-0027] Dlugosch, K. M. , Anderson, S. R. , Braasch, J. , Cang, F. A. , & Gillette, H. D. (2015). The devil is in the details: Genetic variation in introduced populations and its contributions to invasion. Molecular Ecology, 24, 2095–2111. 10.1111/mec.13183 25846825

[eva12913-bib-0028] Dlugosch, K. M. , & Parker, I. M. (2008). Founding events in species invasions: Genetic variation, adaptive evolution, and the role of multiple introductions. Molecular Ecology, 17, 431–449. 10.1111/j.1365-294X.2007.03538.x 17908213

[eva12913-bib-0029] Dowling, D. K. , Friberg, U. , & Lindell, J. (2008). Evolutionary implications of non‐neutral mitochondrial genetic variation. Trends in Ecology & Evolution, 23, 546–554. 10.1016/j.tree.2008.05.011 18722688

[eva12913-bib-0030] Dudaniec, R. Y. , Gardner, M. G. , Donnellan, S. , & Kleindorfer, S. (2008). Genetic variation in the invasive avian parasite, *Philornis downsi* (Diptera, Muscidae) on the Galápagos archipelago. BMC Ecology, 8, 13 10.1186/1472-6785-8-13 18671861 PMC2527555

[eva12913-bib-0031] Estoup, A. , & Guillemaud, T. (2010). Reconstructing routes of invasion using genetic data: Why, how and so what? Molecular Ecology, 19, 4113–4130. 10.1111/j.1365-294X.2010.04773.x 20723048

[eva12913-bib-0032] Excoffier, L. , Smouse, P. E. , & Quattro, J. M. (1992). Analysis of molecular variance inferred from metric distances among DNA haplotypes ‐ application to human mitochondrial‐DNA restriction data. Genetics, 131, 479–491. 1644282 10.1093/genetics/131.2.479 PMC1205020

[eva12913-bib-0033] Foll, M. , & Gaggiotti, O. (2008). A genome‐scan method to identify selected loci appropriate for both dominant and codominant markers: A Bayesian perspective. Genetics, 180, 977 10.1534/genetics.108.092221 18780740 PMC2567396

[eva12913-bib-0034] Fraser, C. I. , Nikula, R. , Ruzzante, D. E. , & Waters, J. M. (2012). Poleward bound: Biological impacts of Southern Hemisphere glaciation. Trends in Ecology & Evolution, 27, 462–471. 10.1016/j.tree.2012.04.011 22658874

[eva12913-bib-0035] Fraser, C. I. , Nikula, R. , Spencer, H. G. , & Waters, J. M. (2009). Kelp genes reveal effects of subantarctic sea ice during the Last Glacial Maximum. Proceedings of the National Academy of Sciences, 106, 3249 10.1073/pnas.0810635106 PMC2651250 19204277

[eva12913-bib-0036] Frenot, Y. , Chown, S. L. , Whinam, J. , Selkirk, P. , Convey, P. , Skotnicki, M. , & Bergstrom, D. M. (2005). Biological invasions in the Antarctic: Extent, impacts and implications. Biological Reviews of the Cambridge Philosophical Society, 80, 45–72. 10.1017/S1464793104006542 15727038

[eva12913-bib-0037] Frenot, Y. , Gloaguen, J. C. , Massé, L. , & Lebouvier, M. (2001). Human activities, ecosystem disturbance and plant invasions in subantarctic Crozet, Kerguelen and Amsterdam Islands. Biological Conservation, 101, 33–50. 10.1016/S0006-3207(01)00052-0

[eva12913-bib-0081] Garnas, J. R. , Auger‐Rozenberg, M.‐A. , Roques, A. , Bertelsmeier, C. , Wingfield, M. J. , Saccaggi, D. L. , … Slippers, B. (2016). Complex patterns of global spread in invasive insects: Eco‐evolutionary and management consequences. Biological Invasions, 18, 935–952. 10.1007/s10530-016-1082-9

[eva12913-bib-0038] Gaston, K. J. , Jones, A. G. , Hänel, C. , & Chown, S. L. (2003). Rates of species introduction to a remote oceanic island. Proceedings of the Royal Society of London. Series B: Biological Sciences, 270, 1091–1098. 12803900 10.1098/rspb.2003.2332 PMC1691340

[eva12913-bib-0039] Geisen, S. , Wall, D. H. , & van der Putten, W. H. (2019). Challenges and opportunities for soil biodiversity in the Anthropocene. Current Biology, 29, R1036–R1044. 10.1016/j.cub.2019.08.007 31593662

[eva12913-bib-0040] Georges, A. , Gruber, B. , Pauly, G. B. , White, D. , Adams, M. , Young, M. J. , … Unmack, P. J. (2018). Genomewide SNP markers breathe new life into phylogeography and species delimitation for the problematic short‐necked turtles (Chelidae: Emydura) of eastern Australia. Molecular Ecology, 27, 5195–5213. 30403418 10.1111/mec.14925

[eva12913-bib-0041] Gillespie, R. G. , Claridge, E. M. , & Roderick, G. K. (2008). Biodiversity dynamics in isolated island communities: Interaction between natural and human‐mediated processes. Molecular Ecology, 17, 45–57. 10.1111/j.1365-294X.2007.03466.x 17727622

[eva12913-bib-0042] Greenslade, P. (2002). Assessing the risk of exotic Collembola invading subantarctic islands: Prioritising quarantine management. Pedobiologia, 46, 338–344. 10.1078/0031-4056-00141

[eva12913-bib-0043] Greenslade, P. , & Convey, P. (2012). Exotic Collembola on subantarctic islands: Pathways, origins and biology. Biological Invasions, 14, 405–417. 10.1007/s10530-011-0086-8

[eva12913-bib-0044] Gruber, B. , Unmack, P. J. , Berry, O. F. , & Georges, A. (2018). dartR : An R package to facilitate analysis of SNP data generated from reduced representation genome sequencing. Molecular Ecology Resources, 18, 691–699. 29266847 10.1111/1755-0998.12745

[eva12913-bib-0045] Halanych, K. M. , & Mahon, A. R. (2018). Challenging dogma concerning biogeographic patterns of Antarctica and the Southern Ocean. Annual Review of Ecology, Evolution, and Systematics, 49(1), 355–378.

[eva12913-bib-0046] Hardouin, E. A. , Chapuis, J.‐L. , Stevens, M. I. , van Vuuren, J. , Quillfeldt, P. , Scavetta, R. J. , … Tautz, D. (2010). House mouse colonization patterns on the sub‐Antarctic Kerguelen Archipelago suggest singular primary invasions and resilience against re‐invasion. BMC Evolutionary Biology, 10, 325 10.1186/1471-2148-10-325 20977744 PMC3087545

[eva12913-bib-0047] Hodgson, D. A. , Graham, A. G. C. , Roberts, S. J. , Bentley, M. J. , Cofaigh, C. Ó. , Verleyen, E. , … Smith, J. A. (2014). Terrestrial and submarine evidence for the extent and timing of the Last Glacial Maximum and the onset of deglaciation on the maritime‐Antarctic and sub‐Antarctic islands. Quaternary Science Reviews, 100, 137–158. 10.1016/j.quascirev.2013.12.001

[eva12913-bib-0048] Houghton, M. , Terauds, A. , Merritt, D. , Driessen, M. , & Shaw, J. (2019). The impacts of non‐native species on the invertebrates of Southern Ocean Islands. Journal of Insect Conservation, 23, 435–452. 10.1007/s10841-019-00147-9

[eva12913-bib-0049] Hudson, J. , Viard, F. , Roby, C. , & Rius, M. (2016). Anthropogenic transport of species across native ranges: Unpredictable genetic and evolutionary consequences. Biology Letters, 12, 20160620 10.1098/rsbl.2016.0620 27729485 PMC5095196

[eva12913-bib-0050] Hughes, K. A. , & Convey, P. (2010). The protection of Antarctic terrestrial ecosystems from inter‐ and intra‐continental transfer of non‐indigenous species by human activities: A review of current systems and practices. Global Environmental Change, 20, 96–112. 10.1016/j.gloenvcha.2009.09.005

[eva12913-bib-0051] Hulme, P. E. (2015). Invasion pathways at a crossroad: Policy and research challenges for managing alien species introductions. Journal of Applied Ecology, 52, 1418–1424. 10.1111/1365-2664.12470

[eva12913-bib-0052] IUCN (2018). Guidelines for invasive species planning and management on islands. Cambridge: IUCN.

[eva12913-bib-0053] Juan, A. , Crespo, M. B. , Cowan, R. S. , Lexer, C. , & Fay, M. F. (2004). Patterns of variability and gene flow in *Medicago citrina*, an endangered endemic of islands in the western Mediterranean, as revealed by amplified fragment length polymorphism (AFLP). Molecular Ecology, 13, 2679–2690. 10.1111/j.1365-294X.2004.02289.x 15315680

[eva12913-bib-0054] Kilian, A. , Wenzl, P. , Huttner, E. , Carling, J. , Xia, L. , Blois, H. , … Uszynski, G. (2012). Diversity Arrays Technology: A generic genome profiling technology on open platforms. Methods in Molecular Biology, 888, 67–89. 22665276 10.1007/978-1-61779-870-2_5

[eva12913-bib-0055] Kolbe, J. J. , Larson, A. , Losos, J. B. , & de Queiroz, K. (2008). Admixture determines genetic diversity and population differentiation in the biological invasion of a lizard species. Biology Letters, 4, 434–437. 10.1098/rsbl.2008.0205 18492644 PMC2610154

[eva12913-bib-0056] Kriwoken, L. K. , & Williamson, J. W. (1993). Hobart, Tasmania: Antarctic and Southern Ocean connections. Polar Record, 29, 93–102. 10.1017/S0032247400023548

[eva12913-bib-0057] Kumar, S. , Stecher, G. , & Tamura, K. (2016). Molecular Evolutionary genetic analysis version 7.0 for bigger datasets. Molecular Biology and Evolution, 33, 1870–1874. 27004904 10.1093/molbev/msw054 PMC8210823

[eva12913-bib-0058] Lavergne, S. , & Molofsky, J. (2007). Increased genetic variation and evolutionary potential drive the success of an invasive grass. Proceedings of the National Academy of Sciences, 104, 3883 10.1073/pnas.0607324104 PMC1805698 17360447

[eva12913-bib-0059] Le Roux, J. , & Wieczorek, A. M. (2009). Molecular systematics and population genetics of biological invasions: Towards a better understanding of invasive species management. Annals of Applied Biology, 154, 1–17. 10.1111/j.1744-7348.2008.00280.x

[eva12913-bib-0060] Le Roux, J. J. , Wieczorek, A. M. , Wright, M. G. , & Tran, C. T. (2007). Super‐genotype: Global monoclonality defies the odds of nature. PLoS ONE, 2, e590. 17611622 10.1371/journal.pone.0000590 PMC1895887

[eva12913-bib-0061] Lebouvier, M. , Laparie, M. , Hullé, M. , Marais, A. , Cozic, Y. , Lalouette, L. , … Renault, D. (2011). The significance of the sub‐Antarctic Kerguelen Islands for the assessment of the vulnerability of native communities to climate change, alien insect invasions and plant viruses. Biological Invasions, 13, 1195–1208. 10.1007/s10530-011-9946-5

[eva12913-bib-0062] Lee, J. E. , & Chown, S. L. (2011). Quantification of intra‐regional propagule movements in the Antarctic. Antarctic Science, 23, 337–342. 10.1017/S0954102011000198

[eva12913-bib-0063] Lenormand, T. (2002). Gene flow and the limits to natural selection. Trends in Ecology & Evolution, 17, 183–189. 10.1016/S0169-5347(02)02497-7

[eva12913-bib-0064] Lloret, F. , Medail, F. , Brundu, G. , Camarda, I. , Moragues, E. , Rita, J. , … Hulme, P. E. (2005). Species attributes and invasion success by alien plants on Mediterranean islands. Journal of Ecology, 93, 512–520. 10.1111/j.1365-2745.2005.00979.x

[eva12913-bib-0065] Losos, J. B. , & Ricklefs, R. E. (2009). Adaptation and diversification on islands. Nature, 457, 830–836. 10.1038/nature07893 19212401

[eva12913-bib-0066] McGaughran, A. , Stevens, M. I. , Hogg, I. D. , & Carapelli, A. (2011). Extreme glacial legacies: A synthesis of the Antarctic springtail phylogeographic record. Insects, 2, 62–82. 10.3390/insects2020062 26467614 PMC4553450

[eva12913-bib-0067] Meirmans, P. G. , & Van Tienderen, P. H. (2004). GENOTYPE and GENODIVE: Two programs for the analysis of genetic diversity of asexual organisms. Molecular Ecology Notes, 4, 792–794. 10.1111/j.1471-8286.2004.00770.x

[eva12913-bib-0068] Moon, K. L. , Chown, S. L. , & Fraser, C. I. (2017). Reconsidering connectivity in the sub‐Antarctic. Biological Reviews, 92, 2164–2181. 10.1111/brv.12327 28371192

[eva12913-bib-0069] Mortimer, E. , van Vuuren, B. J. , Meiklejohn, K. I. , & Chown, S. L. (2012). Phylogeography of a mite, *Halozetes fulvus*, reflects the landscape history of a young volcanic island in the sub‐Antarctic. Biological Journal of the Linnean Society, 105, 131–145. 10.1111/j.1095-8312.2011.01770.x

[eva12913-bib-0070] Moser, D. , Lenzner, B. , Weigelt, P. , Dawson, W. , Kreft, H. , Pergl, J. , … Essl, F. (2018). Remoteness promotes biological invasions on islands worldwide. Proceedings of the National Academy of Sciences, 115, 9270–9275. 10.1073/pnas.1804179115 PMC6140508 30158167

[eva12913-bib-0071] Myburgh, M. , Chown, S. L. , Daniels, S. R. , & van Vuuren, B. J. (2007). Population structure, propagule pressure, and conservation biogeography in the sub‐Antarctic: Lessons from indigenous and invasive springtails. Diversity and Distributions, 13, 143–154. 10.1111/j.1472-4642.2007.00319.x

[eva12913-bib-0072] Nguyen, L.‐T. , Schmidt, H. A. , von Haeseler, A. , & Minh, B. Q. (2014). IQ‐TREE: A fast and effective stochastic algorithm for estimating Maximum‐Likelihood phylogenies. Molecular Biology and Evolution, 32, 268–274. 10.1093/molbev/msu300 25371430 PMC4271533

[eva12913-bib-0073] Novo, M. , Cunha, L. , Maceda‐Veiga, A. , Talavera, J. A. , Hodson, M. E. , Spurgeon, D. , … Kille, P. (2015). Multiple introductions and environmental factors affecting the establishment of invasive species on a volcanic island. Soil Biology and Biochemistry, 85, 89–100. 10.1016/j.soilbio.2015.02.031

[eva12913-bib-0074] Olden, J. D. (2006). Biotic homogenization: A new research agenda for conservation biogeography. Journal of Biogeography, 33, 2027–2039. 10.1111/j.1365-2699.2006.01572.x

[eva12913-bib-0075] Ouisse, T. (2016). Phenotypic and genetic characterisation of the carabid beetle *Merizodus soledadinus* along its invasion gradient at the subantartic Kerguelen Islands. PhD Thesis, Université Rennes

[eva12913-bib-0076] Petit, J. N. , Hoddle, M. S. , Grandgirard, J. , Roderick, G. K. , & Davies, N. (2009). Successful spread of a biocontrol agent reveals a biosecurity failure: Elucidating long distance invasion pathways for *Gonatocerus ashmeadi* in French Polynesia. BioControl, 54, 485–495. 10.1007/s10526-008-9204-7

[eva12913-bib-0077] Petit, R. J. (2004). Biological invasions at the gene level. Diversity and Distributions, 10, 159–165. 10.1111/j.1366-9516.2004.00084.x

[eva12913-bib-0078] Piertney, S. B. , Black, A. , Watt, L. , Christie, D. , Poncet, S. , & Collins, M. A. (2015). Resolving patterns of population genetic and phylogeographic structure to inform control and eradication initiatives for brown rats *Rattus norvegicus* on South Georgia. Journal of Applied Ecology, 53, 332–339.

[eva12913-bib-0079] Porco, D. , Decaëns, T. , Deharveng, L. , James, S. W. , Skarżyński, D. , Erséus, C. , … Hebert, P. D. N. (2013). Biological invasions in soil: DNA barcoding as a monitoring tool in a multiple taxa survey targeting European earthworms and springtails in North America. Biological Invasions, 15, 899–910. 10.1007/s10530-012-0338-2

[eva12913-bib-0080] R Core Team . (2017). R: A language and environment for statistical computing. Vienna: R Foundation for Statistical Computing.

[eva12913-bib-0082] Raj, A. , Stephens, M. , & Pritchard, J. K. (2014). fastSTRUCTURE: Variational inference of population structure in large SNP data sets. Genetics, 197, 573 10.1534/genetics.114.164350 24700103 PMC4063916

[eva12913-bib-0083] Rambaut, A. (2009). FigTree. Retrieved from http://tree.bio.ed.ac.uk/software/figtree

[eva12913-bib-0084] Ratnasingham, S. , & Hebert, P. D. N. (2013). A DNA‐based registry for all animal species: The Barcode Index Number (BIN) system. PLoS ONE, 8, e66213 10.1371/journal.pone.0066213 23861743 PMC3704603

[eva12913-bib-0085] Reaser, J. K. , Meyerson, L. A. , Cronk, Q. , De poorter, M. , Eldrege, L. G. , Green, E. , … Vaiutu, L. (2007). Ecological and socioeconomic impacts of invasive alien species in island ecosystems. Environmental Conservation, 34, 98–111. 10.1017/S0376892907003815

[eva12913-bib-0086] Ricciardi, A. , Blackburn, T. M. , Carlton, J. T. , Dick, J. T. A. , Hulme, P. E. , Iacarella, J. C. , … Aldridge, D. C. (2017). Invasion science: A horizon scan of emerging challenges and opportunities. Trends in Ecology & Evolution, 32, 464–474. 10.1016/j.tree.2017.03.007 28395941

[eva12913-bib-0087] Rieseberg, L. H. , & Burke, J. M. (2001). The biological reality of species: Gene flow, selection, and collective evolution. Taxon, 50, 47–67. 10.2307/1224511

[eva12913-bib-0089] Seehausen, O. (2006). Conservation: Losing biodiversity by reverse speciation. Current Biology, 16, R334–R337. 10.1016/j.cub.2006.03.080 16682344

[eva12913-bib-0090] Sethuraman, A. , Gonzalez, N. M. , Grenier, C. E. , Kansagra, K. S. , Mey, K. K. , Nunez‐Zavala, S. B. , … Wulf, G. K. (2019). Continued misuse of multiple testing correction methods in population genetics‐ a wake‐up call? Molecular Ecology Resources, 19, 23–26. 10.1111/1755-0998.12969 30701708

[eva12913-bib-0091] Slatkin, M. (1987). Gene flow and the geographic structure of natural populations. Science, 236, 787 10.1126/science.3576198 3576198

[eva12913-bib-0092] Stevens, M. I. , Greenslade, P. , Hogg, I. D. , & Sunnucks, P. (2006). Southern hemisphere springtails: Could any have survived glaciation of Antarctica? Molecular Biology and Evolution, 23, 874–882. 10.1093/molbev/msj073 16326749

[eva12913-bib-0093] Stewart, J. E. , Espiner, S. , Liggett, D. , & Taylor, Z. (2017). The forgotten islands: Monitoring tourist numbers and managing tourism impacts on New Zealand’s Subantarctic Islands. Resources, 6, 38 10.3390/resources6030038

[eva12913-bib-0094] Toews, D. P. L. , & Brelsford, A. (2012). The biogeography of mitochondrial and nuclear discordance in animals. Molecular Ecology, 21, 3907–3930. 10.1111/j.1365-294X.2012.05664.x 22738314

[eva12913-bib-0095] Tønnessen, J. N. , & Johnsen, A. O. (1982). The history of modern whaling. Los Angeles: University of California Press.

[eva12913-bib-0096] UNESCO (2019). French Austral lands and seas. World Heritage List. Retrieved from https://https:/whc.unesco.org/en/list/1603

[eva12913-bib-0097] Van Der Wurff, A. W. G. , Isaaks, J. A. , Ernsting, G. , & Van Straalen, N. M. (2003). Population substructures in the soil invertebrate *Orchesella cincta*, as revealed by microsatellite and TE‐AFLP markers. Molecular Ecology, 12, 1349–1359. 10.1046/j.1365-294X.2003.01811.x 12755866

[eva12913-bib-0098] van Vuuren, B. J. , Lee, J. E. , Convey, P. , & Chown, S. L. (2018). Conservation implications of spatial genetic structure in two species of oribatid mites from the Antarctic Peninsula and the Scotia Arc. Antarctic Science, 30(2), 105–114. 10.1017/S0954102017000529

[eva12913-bib-0099] Vandergast, A. G. , Gillespie, R. G. , & Roderick, G. K. (2004). Influence of volcanic activity on the population genetic structure of Hawaiian Tetragnatha spiders: Fragmentation, rapid population growth and the potential for accelerated evolution. Molecular Ecology, 13, 1729–1743. 10.1111/j.1365-294X.2004.02179.x 15189199

[eva12913-bib-0100] von Saltzwedel, H. , Scheu, S. , & Schaefer, I. (2016). Founder events and pre‐glacial divergences shape the genetic structure of European Collembola species. BMC Evolutionary Biology, 16, 148 10.1186/s12862-016-0719-8 27423184 PMC4947257

[eva12913-bib-0101] Wagner, N. K. , Ochocki, B. M. , Crawford, K. M. , Compagnoni, A. , & Miller, T. E. X. (2017). Genetic mixture of multiple source populations accelerates invasive range expansion. Journal of Animal Ecology, 86, 21–34. 10.1111/1365-2656.12567 27363388

[eva12913-bib-0102] Walton, D. H. , & Thomas, J. (2018). Cruise report ‐ Antarctic Circumnavigation Expedition (ACE) 20th December 2016–19th March 2017 (Version 1.0), Zenodo, 10.5281/zenodo.1443511

[eva12913-bib-0103] Weiner, J. (2017). pca3d: Three dimensional PCA plots. R Package Version, 10. Available at https://CRAN.R%2013project.org/package=pca3d.

[eva12913-bib-0104] Weir, B. S. , & Cockerham, C. C. (1984). Estimating F‐statistics for the analysis of population structure. Evolution, 38, 1358–1370. 28563791 10.1111/j.1558-5646.1984.tb05657.x

[eva12913-bib-0105] White, T. , van der Ende, J. , & Nichols, T. E. (2019). Beyond Bonferroni revisited: Concerns over inflated false positive research findings in the fields of conservation genetics, biology, and medicine. Conservation Genetics, 20(4), 927–937. 10.1007/s10592-019-01178-0

[eva12913-bib-0106] Wouw, M. , Dijk, P. V. , & Huiskes, A. H. L. (2008). Regional genetic diversity patterns in Antarctic hairgrass (*Deschampsia antarctica* Desv.). Journal of Biogeography, 35, 365–376.

