# Supplementary material for: Springtail phylogeography highlights biosecurity risks of repeated invasions and intraregional transfers among remote islands
Source: Evol Appl. 2020 Feb 12;13(5):960–73. doi: 10.1111/eva.12913 (PMC7232766; doi:10.1111/eva.12913)

Supporting Information

Baird, H. P., Moon, K. L., Janion-Scheepers, C. and Chown, S. L. (2020) Springtail phylogeography highlights biosecurity risks of repeated invasions and intraregional transfers among remote islands. *Evolutionary Applications*.

Table of Contents

Supporting Information S1	2
Tables S1.1 & S1.2	4
Tables S2.1 & S2.2	5
Tables S3.1 & S3.2	6
Table S4	10
Table S5	11
Table S6	11
Table S7	12
Table S8	12
Table S9	13
Table S10	13
Table S11	14
Table S12	14
Figure S1	15
Figure S2	16
Figure S3	17
Figure S4	18
Figure S5	19
Figure S6	20

Supporting Information S1

DArT-Seq SNP library preparation and quality filtering

Genomic DNA extraction and sequencing of the springtails *Hypogastrura viatica* and *Tullbergia bisetosa* was carried out at Diversity Arrays Technology (DArT) in Canberra, Australia. DArT employ DArT-SeqTM sequencing technology, which is a reduced representation next-generation sequencing method similar to double-digest restriction associated DNA sequencing (ddRAD). Following preliminary testing, the enzyme combination *Pts*I - *Hpa*II was chosen for both springtail species for the double-digestion of genomic DNA. Digestion/ligation reactions (see method described in Kilian et al., 2012) were then used to ligate specific adaptors to both restriction enzyme overhangs. These adaptors facilitate sample identification (using unique barcodes) and Illumina sequencing, in a manner similar to that described in Elshire et al. (2011). Fragments were amplified in a PCR using the following conditions: 1 min at 94°C for denaturation; 30 cycles each consisting of 20 s at 94°C, 30 s at 58°C, 45 s at 72°C, followed by a final extension of 7 min at 72°C. Equimolar amounts of amplified product for each sample were then pooled and sequenced using an Illumina Hiseq2500.

Raw Illumina data were processed using a proprietary DArT pipeline. Sequences were processed to filter out bad quality reads (minimum read Phred score of 10), with more stringent filters applied to barcode regions of the fragments (minimum Phred score of 30) to ensure sequences would be assigned to samples reliably. Fragments were trimmed to 69 bp, and a second proprietary DArT pipeline (DArTsoft14) was used to call candidate SNP markers and conduct initial filtering. During this phase, SNPs were filtered for >90% reproducibility (informed by technical sample replicates), 20% call rate and a minimum read depth of 5.

The raw SNP data files generated by DArT for each species were imported as genlight objects into R v.3.4.3 for additional quality filtering using the 'dartR' package v.1.0.5 (Gruber et al., 2018). While the DArTsoft14 pipeline ensured that SNPs had a call rate of at least 20% (i.e. were present in at least 20% of individuals), call rates in the genomic literature are typically higher, ranging from 50% to 100%. There is growing evidence that less stringent filtering (lower call rates) provides greater phylogenetic resolution despite incurring more missing data (e.g. Díaz-Arce et al., 2016; Wagner et al., 2013), therefore, we chose to filter our SNPs to a moderate call rate of 80%. Secondary reads (those called from the same fragment as another SNP) were excluded to minimize genetic linkage, while further screening ensured that the minor allele frequency was ≥2% globally (across all geographic sites) or ≥20% locally (within a site). Following this, individuals with ≥50% missing data were removed and any loci that became monomorphic as a result were also removed. The SNP dataset sizes at each filtering step are provided in Table S4.

References

Díaz-Arce, N., Arrizabalaga, H., Murua, H., Irigoien, X. & Rodríguez-Ezpeleta, N. (2016). RAD-seq derived genome-wide nuclear markers resolve the phylogeny of tunas. *Molecular Phylogenetics and Evolution*, *102*, 202-207.

Elshire, R. J., Glaubitz, J. C., Sun, Q., Poland, J. A., Kawamoto, K., Buckler, E. S. & Mitchell, S. E. (2011). A robust, simple genotyping-by-sequencing (GBS) approach for high diversity species. *PLoS ONE*, *6*, e19379.

Gruber, B., Unmack, P. J., Berry, O. F. & Georges, A. (2018). dartR : An R package to facilitate analysis of SNP data generated from reduced representation genome sequencing. *Molecular Ecology Resources*, *18*, 691-699.

Kilian, A., Wenzl, P., Huttner, E., Carling, J., Xia, L., Blois, H., . . . Uszynski, G. (2012). Diversity Arrays Technology: A generic genome profiling technology on open platforms. *Methods in Molecular Biology, 888,* 67-89.

Wagner, C. E., Keller, I., Wittwer, S., Selz, O. M., Mwaiko, S., Greuter, L., . . . Seehausen, O. (2013). Genome-wide RAD sequence data provide unprecedented resolution of species boundaries and relationships in the Lake Victoria cichlid adaptive radiation. *Molecular Ecology*, *22*, 787-798.

Table S1.1 Sample collection details for the indigenous springtail *Tullbergia bisetosa*. The island pertaining to each site is provided in the first column. Sites within an island discerned by a letter suffix (1a, b, c..) are separated by <5 km; sites within an island discerned by a number suffix (1, 2…) are separated by >10 km. *N* represents the final sample size after individuals with >50% missing SNP data were removed.

	Site	*N*	Latitude	Longitude	Collection date
Macquarie	MQ-1a	17	-54.49699	158.94154	Mar 2018
	MQ-1b	10	-54.50354	158.93243	Mar 2018
	MQ-2	19	-54.67930	158.82564	Dec 2010
Heard	HD-1a	4	-53.06385	73.65347	Jan 2004
	HD-1b	13	-53.06141	73.65297	Jan 2004
Marion	MR-1a	16	-46.88515	37.86816	Dec 2016
	MR-1b	23	-46.87688	37.86063	Dec 2016
	MR-1c	17	-46.89870	37.89326	Dec 2016
South Georgia	SG-1a	20	-54.17773	-37.62131	Jan 2015
	SG-1b	19	-54.18230	-37.62329	Jan 2015
	SG-2	20	-54.01540	-38.01136	Jan 2015

Table S1.2 Sample collection details for the invasive springtail *Hypogastrura viatica*. The island pertaining to each site is provided in the first column. Sites within an island discerned by a letter suffix (1a, b, c..) are separated by <5 km; sites within an island discerned by a number suffix (1, 2…) are separated by >10 km. *N* represents the final sample size after individuals with >50% missing SNP data were removed.

	Site	*N*	Latitude	Longitude	Collection date
Macquarie	MQ-H-1a	16	-54.49863	158.94092	Mar 2018
	MQ-H-1b	22	-54.50357	158.93243	Mar 2017
	MQ-H-1c	14	-54.50412	158.93105	Mar 2018
	MQ-H-2	21	-54.63040	158.89777	Mar 2017
Kerguelen	KR-1a	19	-49.35310	70.07726	Jan 2017
	KR-1b	19	-49.35410	70.07931	Jan 2017
Possession	PS-1a	17	-46.39116	51.80608	Dec 2016
	PS-1b	19	-46.38979	51.80292	Dec 2016
South Georgia	SG-H-1	20	-54.23788	-36.64027	Jan 2014
	SG-H-2	11	-54.15762	-36.81538	Jan 2014

Table S2.1 Matrix of shortest geographic distances (in km, to the nearest 10 m) between each pair of sites for *Tullbergia bisetosa.* Distances were calculated using a coordinate-based distance calculator at https://gps-coordinates.org/distance-between-coordinates.php and verified using Google Maps.

	MQ-1a	MQ-1b	MQ-2	HD-1a	HD-1b	MR-1a	MR-1b	MR-1c	SG-1a	SG-1b	SG-2
**MQ-1a**	-										
**MQ-1b**	0.94	-									
**MQ-2**	21.60	19.54	-								
**HD-1a**	5249.02	5242.49	5231.23	-							
**HD-1b**	5249.21	5242.68	5231.42	0.27	-						
**MR-1a**	7459.23	7453.90	7438.84	2619.55	2619.51	-					
**MR-1b**	7460.31	7454.99	7439.92	2620.49	2620.46	1.08	-				
**MR-1c**	7456.93	7451.61	7436.54	2617.16	2617.12	2.43	3.47	-			
**SG-1a**	7835.92	7836.53	7817.29	6519.88	6520.07	5137.37	5137.49	5137.93	-		
**SG-1b**	7835.40	7836.00	7816.76	6519.58	6519.77	5137.28	5137.40	5137.85	0.52	-	
**SG-2**	7849.17	7848.56	7830.58	6550.17	6550.35	5167.92	5168.04	5168.48	31.19	31.38	-

Table S2.2 Matrix of shortest geographic distances (in km, to the nearest 10 m) between each pair of sites for *Hypogastrura viatica.* Distances were calculated using a coordinate-based distance calculator at https://gps-coordinates.org/distance-between-coordinates.php and verified using Google Maps.

	MQ-H-1a	MQ-H-1b	MQ-H-1c	MQ-H-2	KR-1a	KR-1b	PS-1a	PS-1b	SG-H-1	SG-H-2
**MQ-H-1a**	-									
**MQ-H-1b**	0.78	-								
**MQ-H-1c**	0.88	0.11	-							
**MQ-H-2**	14.91	14.28	14.21	-						
**KR-1a**	5704.71	5703.95	5703.85	5694.33	-					
**KR-1b**	5704.53	5703.77	5703.66	5694.14	1.89	-				
**PS-1a**	6879.77	6878.99	6878.89	6867.96	1398.26	1398.42	-			
**PS-1b**	6880.04	6879.27	6879.16	6868.23	1398.54	1398.70	0.29	-		
**SG-H-1**	7840.04	7839.59	7839.55	7826.06	6626.20	6626.21	5919.96	5919.88	-	
**SG-H-2**	7846.95	7846.50	7846.45	7832.97	6640.60	6640.61	5934.31	534.23	14.47	-

Table S3.1 Accession details for COI sequences ≥ 500bp retrieved for the springtail *Tullbergia bisetosa* from BOLD BIN AAA6992 in January 2019. The sequence identifier on BOLD is provided, except where a sequence was mined from Genbank (via the BIN search), in which case the Genbank identifier is provided.

Sample origin (country/island)	Sequence ID (BOLD)	Sequence ID (Genbank)
Chile		KP027077
Heard Island	COLAN271-09	
Heard Island	COLAN273-09	
Heard Island	COLAN274-09	
Heard Island	COLAN275-09	
Heard Island	COLAN276-09	
Heard Island	COLAN277-09	
Heard Island	COLAN317-09	
Heard Island	COLAN319-09	
Heard Island	COLAN320-09	
Macquarie Island	COLNZ422-09	
Macquarie Island	COLNZ424-09	
Macquarie Island	COLNZ426-09	
Macquarie Island	COLNZ427-09	
Macquarie Island	COLNZ429-09	
Macquarie Island	COLNZ430-09	
Macquarie Island	COLNZ431-09	
Macquarie Island	COLMU339-16	
Macquarie Island	COLMU340-16	
Macquarie Island	COLMU577-16	
Macquarie Island	COLMU578-16	
Macquarie Island	COLMU579-16	
Macquarie Island	COLMU580-16	
Macquarie Island	COLMU581-16	
Macquarie Island	COLMU582-16	
Marion Island		HQ592668
Marion Island		HQ592667
Marion Island		HQ592666
Marion Island		HQ592665
Marion Island		HQ592664
Marion Island		DQ147451
Marion Island		DQ147450
Marion Island		DQ147449
Marion Island		DQ147448
Marion Island		DQ147447
Marion Island		DQ147443
Marion Island		DQ147442
Marion Island		DQ147440
Marion Island		DQ147439
Marion Island		DQ147437
Marion Island		DQ147436
Marion Island		DQ147435
Marion Island		DQ147434
Marion Island		DQ147433
Marion Island		DQ147432
Marion Island		DQ147431
Marion Island		DQ147430
Marion Island		DQ147428

Marion Island		DQ147427
Marion Island		DQ147425
Marion Island		DQ147424
Marion Island		DQ147423
Marion Island		DQ147422
Marion Island		DQ147421
Marion Island		DQ147420
Marion Island		DQ147419
Marion Island		DQ147418
Marion Island		DQ147417
Marion Island		DQ147416
Marion Island		DQ147415
Marion Island		DQ147414
Marion Island		DQ147413
Marion Island		DQ147412
Marion Island	COLMU1094-17	
Marion Island	COLMU1095-17	
Marion Island	COLMU1096-17	
Marion Island	COLMU1097-17	
Marion Island	COLMU1098-17	
Marion Island	COLMU1099-17	
South Georgia	COLMU438-16	

Table S3.2 Accession details for COI sequences ≥ 500bp retrieved for the springtail *Hypogastrura viatica* from BOLD BIN AAA4806 in January 2019. The sequence identifier on BOLD is provided, except where a sequence was mined from Genbank (via the BIN search), in which case the Genbank identifier is provided.

Sample origin (country/island)	Sequence ID (BOLD)	Sequence ID (Genbank)
Canada	MHCLM105-07	
Canada	MHCLM194-08	
Canada	MHCLM196-08	
Canada	MHCLM197-08	
Canada	MHCLM217-08	
Canada	MHCLM218-08	
Canada	MHCLM219-08	
Canada	MHCLM220-08	
Canada	MHCLM222-08	
Canada	MHCLM361-08	
Canada	MHCLM487-08	
Canada	MHCLM536-08	
Canada	MHCLM537-08	
Canada	MHCLM538-08	
Canada	MHCLM540-08	
Canada	MHCLM573-08	
Canada	MHCLM574-08	
Canada	MHCLM575-08	
Canada	CONAB787-10	
Canada	CONAB788-10	
Canada	CONAB789-10	
Canada	CONAB790-10	
Canada	CONAB791-10	
Canada	CHIP039-12	
Canada	CHIP040-12	
Canada	CHIP041-12	
Denmark	COLLG215-10	
Denmark	COLLG216-10	
Denmark	COLLG217-10	
Denmark	COLLG218-10	
Denmark	COLLG219-10	
Denmark	COLLG220-10	
Greenland		DQ309571
Possession Island	COLMU1153-17	
Possession Island	COLMU1154-17	
Possession Island	COLMU1155-17	
Possession Island	COLMU1156-17	
Possession Island	COLMU1157-17	
Possession Island	COLMU1158-17	
Possession Island	COLMU846-17	
Kerguelen Island	COLMU1242-17	
Kerguelen Island	COLMU1243-17	
Kerguelen Island	COLMU1244-17	
Kerguelen Island	COLMU1245-17	
Kerguelen Island	COLMU1246-17	
Kerguelen Island	COLMU1247-17	
Kerguelen Island	COLMU847-17	
Kerguelen Island	COLMU848-17	

Kerguelen Island	COLMU849-17	
Macquarie Island	COLMU1311-17	
Macquarie Island	COLMU1312-17	
Macquarie Island	COLMU1313-17	
Macquarie Island	COLMU1314-17	
Macquarie Island	COLMU1315-17	
Macquarie Island	COLMU1316-17	
Macquarie Island	COLMU1326-17	
Macquarie Island	COLMU1327-17	
Macquarie Island	COLMU1328-17	
Macquarie Island	COLMU1329-17	
Macquarie Island	COLMU601-16	
Macquarie Island	COLMU603-16	
Macquarie Island	COLMU604-16	
Macquarie Island	COLMU605-16	
Macquarie Island	COLMU606-16	
Macquarie Island	COLMU842-17	
Macquarie Island	COLMU843-17	
Macquarie Island	COLMU844-17	
Macquarie Island	COLMU845-17	
Macquarie Island	COLMU076-15	
Macquarie Island	COLMU077-15	
Macquarie Island	COLMU078-15	
Macquarie Island		HQ732066
Macquarie Island		HQ732067
Macquarie Island		HQ732068
Macquarie Island		HQ732069
New Zealand	COLNZ049-08	
New Zealand	COLNZ050-08	
New Zealand	COLNZ051-08	
New Zealand	COLNZ052-08	
New Zealand	COLNZ053-08	
New Zealand	COLNZ265-08	
New Zealand	COLNZ266-08	
New Zealand	COLNZ268-08	
New Zealand		DQ309570
New Zealand		HQ732070
Norway	DAREK019-09	
South Africa	COLSA776-11	
South Africa	COLSA777-11	
South Africa	COLSA778-11	
South Georgia	COLMU174-15	
South Georgia	COLMU175-15	
South Georgia	COLMU176-15	
South Georgia	COLMU189-15	
South Georgia	COLMU190-15	

Table S4 Number of genome-wide SNPs at each stage of quality filtering for the springtails *Tullbergia bisetosa* and *Hypogastrura viatica*. The final two steps (in grey) were only carried out for the conservative dataset.
MAF: minor allele frequency; HWE: Hardy-Weinberg Equilibrium
*Note that monomorphic loci were identified once individuals with >50% missing data had been removed from the dataset

	T. bisetosa	*H. viatica*
Reproducibility >90% + read depth ≥5	39,847	37,803
Call rate ≥80%	11,897	11,172
Secondary reads removed	7,432	8,117
MAF ≥2% globally or ≥20% locally	5,715	7,277
Monomorphic loci removed*	5,680	7,275
Loci putatively under selection removed	5,557	7,117
Loci significantly violating HWE removed	4,914	5,281

Table S5 Genetic diversity statistics for populations of *Tullbergia bisetosa*, based on a conservative dataset of 4,914 SNPs (excluding loci putatively under selection or violating Hardy Weinberg Equilibrium: see main text). Genetic diversity indices from South Georgia were found to be significantly different to all other islands ($p < 0.05$) and are shown in bold.
N = sample size; H_O = observed heterozygosity; H_E = expected heterozygosity; F_{IS} = inbreeding coefficient.

	Site	H_O	H_E	F_{IS}
Macquarie	MQ-1a	0.044	0.062	0.29
	MQ-1b	0.049	0.064	0.242
	MQ-2	0.056	0.065	0.138
Heard	HD-1a	0.058	0.072	0.196
	HD-1b	0.051	0.066	0.227
Marion	MR-1a	0.045	0.067	0.325
	MR-1b	0.069	0.097	0.288
	MR-1c	0.056	0.089	0.369
Sth Georgia	SG-1a	**0.106**	**0.148**	0.285
	SG-1b	**0.111**	**0.144**	0.229
	SG-2	**0.111**	**0.152**	0.266
	Global	0.069	0.094	0.268

Table S6 AMOVA outcomes for *Tullbergia bisetosa* based on a conservative dataset of 4,914 SNPs (excluding loci putatively under selection or violating Hardy Weinberg Equilibrium: see main text).
SS = sum of squares; % var = percent of total variation

	SS	% var	*F*-statistic
Among island	70129.53	40.8	0.41
Among site within island	16888.23	10.5	0.18
Among samples within site	62361.57	12.9	0.27
Within samples	38598.50	35.8	0.64

Table S7 Genetic diversity statistics for populations of *Hypogastrura viatica*, based on a conservative dataset of 5,281 SNPs (excluding loci putatively under selection or violating Hardy Weinberg Equilibrium: see main text).
N = sample size; H_O = observed heterozygosity; H_E = expected heterozygosity; F_{IS} = inbreeding coefficient.

	Site	H_O	H_E	F_{IS}
Macquarie	MQ-H-1a	0.140	0.206	0.321
	MQ-H-1b	0.143	0.192	0.253
	MQ-H-1c	0.150	0.203	0.261
	MQ-H-2	0.132	0.204	0.354
Kerguelen	KR-1a	0.150	0.206	0.276
	KR-1b	0.145	0.205	0.293
Possession	PS-1a	0.146	0.199	0.264
	PS-1b	0.149	0.202	0.260
Sth Georgia	SG-H-1	0.140	0.206	0.321
	SG-H-2	0.143	0.192	0.253
	Global	0.140	0.202	0.303

Table S8 AMOVA outcomes for *Hypogastrura viatica* based on a conservative dataset of 5,281 SNPs (excluding loci putatively under selection or violating Hardy Weinberg Equilibrium: see main text).
SS = sum of squares; % var = percent of total variation

	SS	% var	*F*-statistic
Among island	17997.50	9.4	0.09
Among site within island	6823.71	2.1	0.02
Among samples within site	114977.07	23.6	0.27
Within samples	70614.00	65.0	0.35

Table S9 Matrix of pairwise F_{ST} estimates for *Tullbergia bisetosa* populations, based on 5,680 SNPs. F_{ST} estimates are shown below the diagonal; uncorrected p-values (determined by 5,000 permutations) are shown above the diagonal.

	**MQ-1a**	**MQ-1b**	**MQ-2**	**HD-1a**	**HD-1b**	**MR-1a**	**MR-1b**	**MR-1c**	**SG-1a**	**SG-1b**	**SG-2**
**MQ-1a**	-	0.000	0.000	0.000	0.000	0.000	0.000	0.000	0.000	0.000	0.000
**MQ-1b**	0.053	-	0.000	0.000	0.000	0.000	0.000	0.000	0.000	0.000	0.000
**MQ-2**	0.077	0.077	-	0.000	0.000	0.000	0.000	0.000	0.000	0.000	0.000
**HD-1a**	0.574	0.569	0.565	-	0.003	0.000	0.001	0.000	0.000	0.000	0.000
**HD-1b**	0.577	0.573	0.567	0.027	-	0.000	0.000	0.000	0.000	0.000	0.000
**MR-1a**	0.669	0.666	0.662	0.667	0.677	-	0.000	0.000	0.000	0.000	0.000
**MR-1b**	0.628	0.616	0.628	0.604	0.632	0.346	-	0.000	0.000	0.000	0.000
**MR-1c**	0.650	0.640	0.648	0.626	0.654	0.331	0.280	-	0.000	0.000	0.000
**SG-1a**	0.576	0.548	0.578	0.506	0.562	0.585	0.584	0.581	-	0.000	0.000
**SG-1b**	0.582	0.555	0.584	0.516	0.571	0.592	0.587	0.586	0.087	-	0.000
**SG-2**	0.601	0.574	0.605	0.536	0.588	0.605	0.601	0.601	0.271	0.312	-

Table S10 Number of SNPs (out of 5,680) with fixed allelic differences for each pair of populations of *Tullbergia bisetosa*. Values found to be statistically above the false positive rate (determined by 5,000 simulations) are indicated in bold.

	**MQ-1a**	**MQ-1b**	**MQ-2**	**HD-1a**	**HD-1b**	**MR-1a**	**MR-1b**	**MR-1c**	**SG-1a**	**SG-1b**	**SG-2**
**MQ-1a**	-										
**MQ-1b**	0	-									
**MQ-2**	0	0	-								
**HD-1a**	**197**	**223**	**193**	-							
**HD-1b**	**150**	**173**	**147**	2	-						
**MR-1a**	**296**	**323**	**273**	**411**	**347**	-					
**MR-1b**	**267**	**294**	**246**	**375**	**319**	**23**	-				
**MR-1c**	**274**	**302**	**253**	**385**	**326**	**9**	**11**	-			
**SG-1a**	**194**	**213**	**184**	**271**	**237**	**238**	**209**	**221**	-		
**SG-1b**	**205**	**224**	**194**	**279**	**244**	**256**	**223**	**236**	0	-	
**SG-2**	**238**	**255**	**221**	**311**	**272**	**280**	**239**	**259**	**17**	**23**	-

Table S11 Matrix of pairwise F_{ST} estimates for *Hypogastrura viatica* populations, based on 7,275 SNPs. F_{ST} estimates are shown below the diagonal; uncorrected p-values (determined by 5,000 permutations) are shown above the diagonal.

	MQ-H-1a	MQ-H-1b	MQ-H-1c	MQ-H-2	KR-1a	KR-1b	PS-1a	PS-1b	SG-H-1	SG-H-2
**MQ-H-1a**	-	0.058	0.000	0.000	0.000	0.000	0.000	0.000	0.000	0.000
**MQ-H-1b**	0.002	-	0.029	0.000	0.000	0.000	0.000	0.000	0.000	0.000
**MQ-H-1c**	0.005	0.002	-	0.000	0.000	0.000	0.000	0.000	0.000	0.000
**MQ-H-2**	0.046	0.042	0.043	-	0.000	0.000	0.000	0.000	0.000	0.000
**KR-1a**	0.119	0.117	0.117	0.148	-	0.009	0.000	0.000	0.000	0.000
**KR-1b**	0.118	0.116	0.116	0.149	0.003	-	0.000	0.000	0.000	0.000
**PS-1a**	0.139	0.134	0.138	0.169	0.034	0.029	-	0.018	0.000	0.000
**PS-1b**	0.135	0.131	0.133	0.164	0.032	0.025	0.004	-	0.000	0.000
**SG-H-1**	0.166	0.164	0.167	0.195	0.203	0.201	0.221	0.217	-	0.000
**SG-H-2**	0.158	0.154	0.159	0.189	0.190	0.189	0.208	0.204	0.088	-

Table S12 Number of SNPs (out of 7,275) with fixed allelic differences for each pair of populations of *Hypogastrura viatica*. None of the values were found to be statistically significant, based on a false positive rate determined by 5,000 simulations.

	MQ-H-1a	MQ-H-1b	MQ-H-1c	MQ-H-2	KR-1a	KR-1b	PS-1a	PS-1b	SG-H-1	SG-H-2
**MQ-H-1a**	-									
**MQ-H-1b**	0	-								
**MQ-H-1c**	0	0	-							
**MQ-H-2**	0	0	0	-						
**KR-1a**	0	0	0	0	-					
**KR-1b**	0	1	1	0	0	-				
**PS-1a**	0	0	0	0	0	0	-			
**PS-1b**	0	0	0	1	0	0	0	-		
**SG-H-1**	0	0	0	1	1	1	1	2	-	
**SG-H-2**	2	1	3	2	2	2	3	2	0	-

Figure S1 PCoA for *Tullbergia bisetosa* based on a conservative dataset of 4,914 SNPs (excluding loci putatively under selection or violating Hardy Weinberg Equilibrium: see main text). The first two principal coordinate axes explain 44.7% of genetic variation. Individuals are coloured by population as shown.

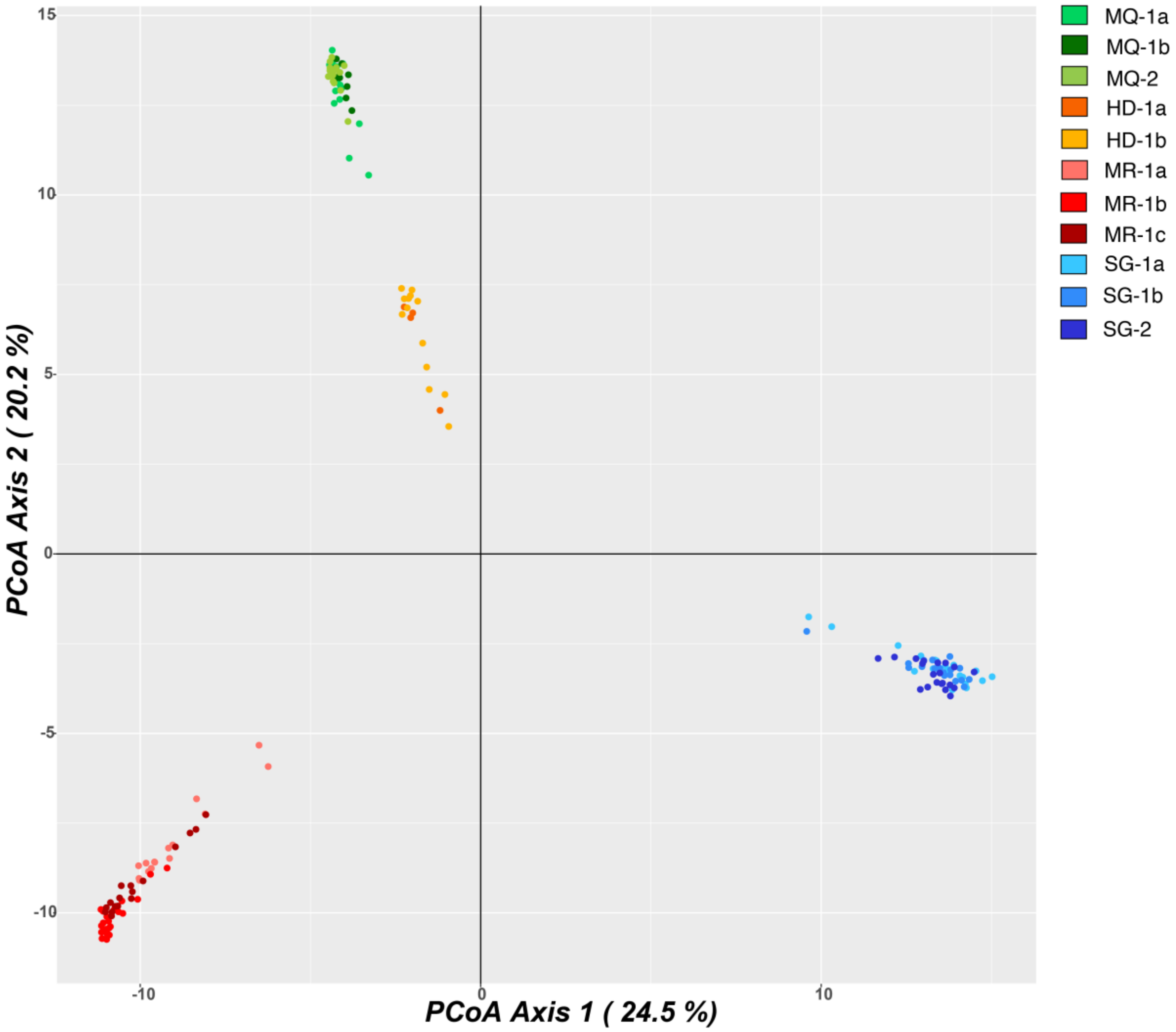

Figure S2 PCoA for *Hypogastrura viatica* based on a conservative dataset of 5,281 SNPs (excluding loci putatively under selection or violating Hardy Weinberg Equilibrium: see main text). The first two principal coordinate axes explain 12.5% of genetic variation. Individuals are coloured by population as shown.

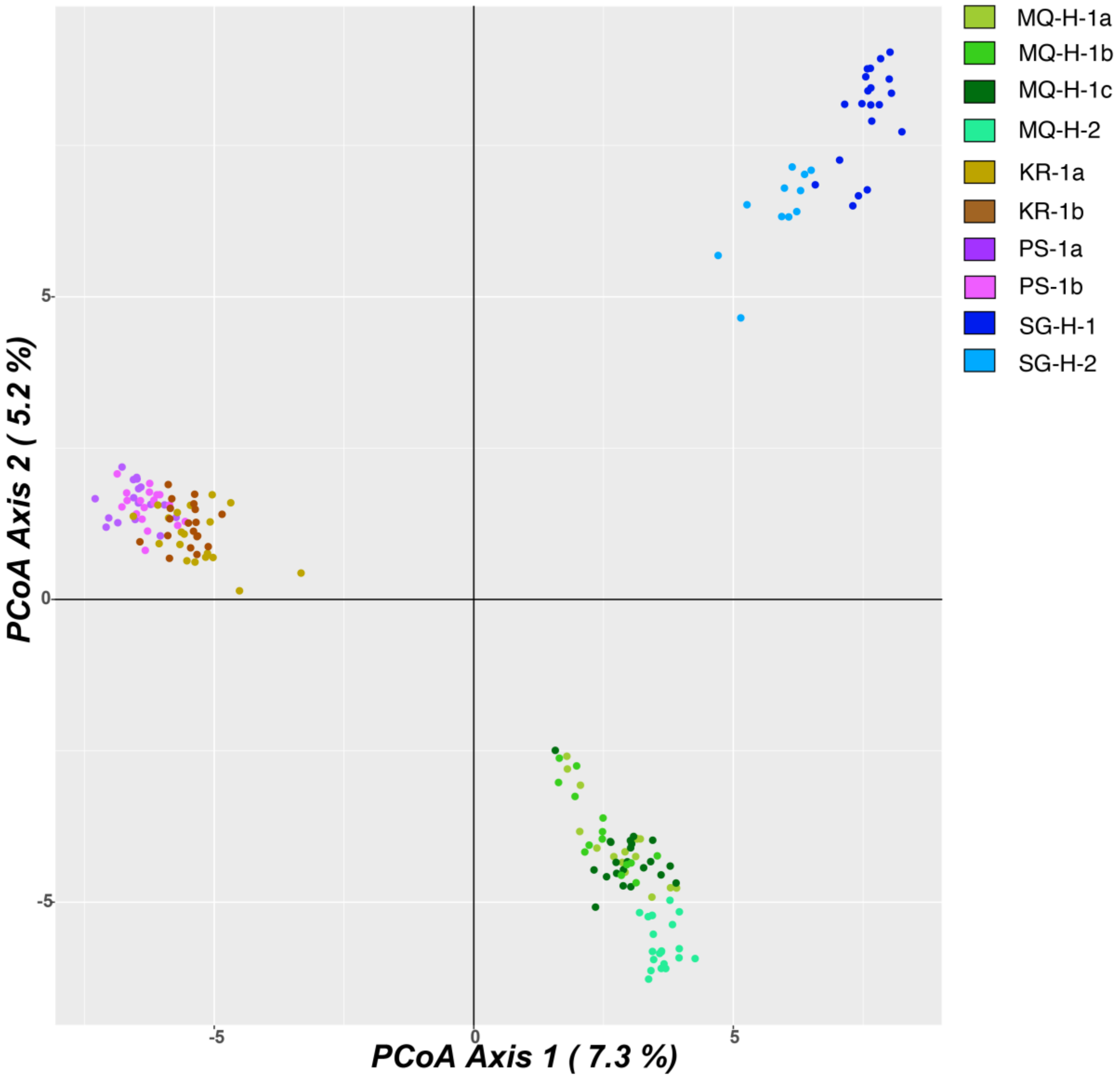

Figure S3 3D PCA for *Tullbergia bisetosa* based on 5,680 SNPs. The three axes shown explain 49.1% of genetic variation. Individuals are coloured by population.

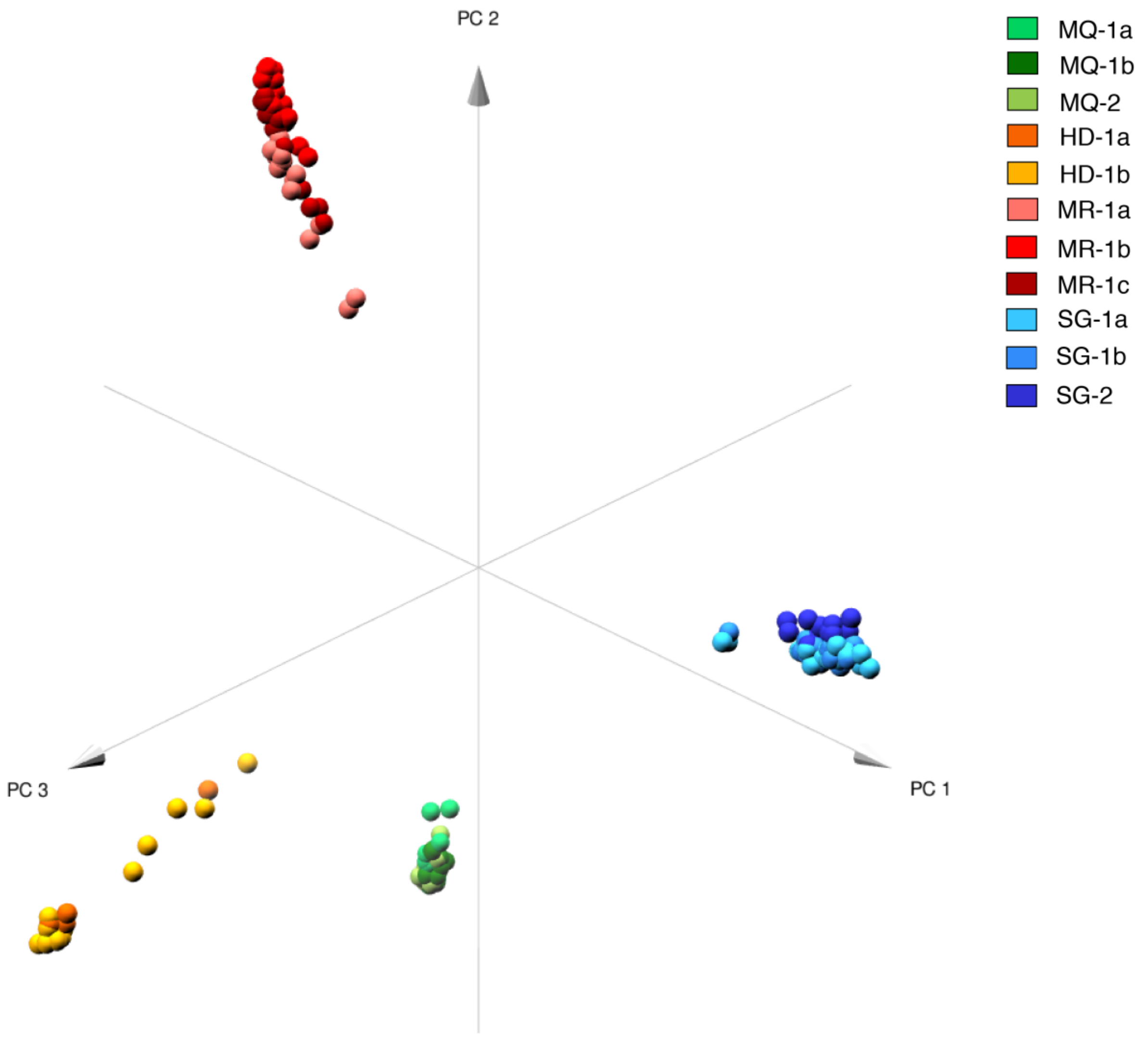

Figure S4 fastSTRUCTURE plot of *K*=7 identified clusters for *Tullbergia bisetosa*, based on 5,680 SNPs. Each vertical bar shows the degree of membership of an individual to one of seven clusters, represented by colours. The geographic source populations for individuals are indicated below the plot.

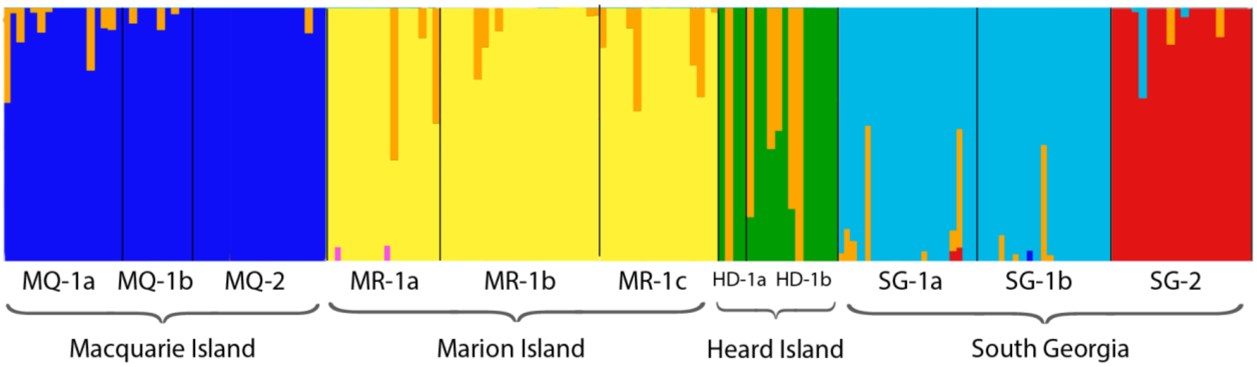

Figure S5 3D PCA for *Hypogastrura viatica* based on 7,275 SNPs. The three axes shown explain 14.1% of genetic variation. Individuals are coloured by population.

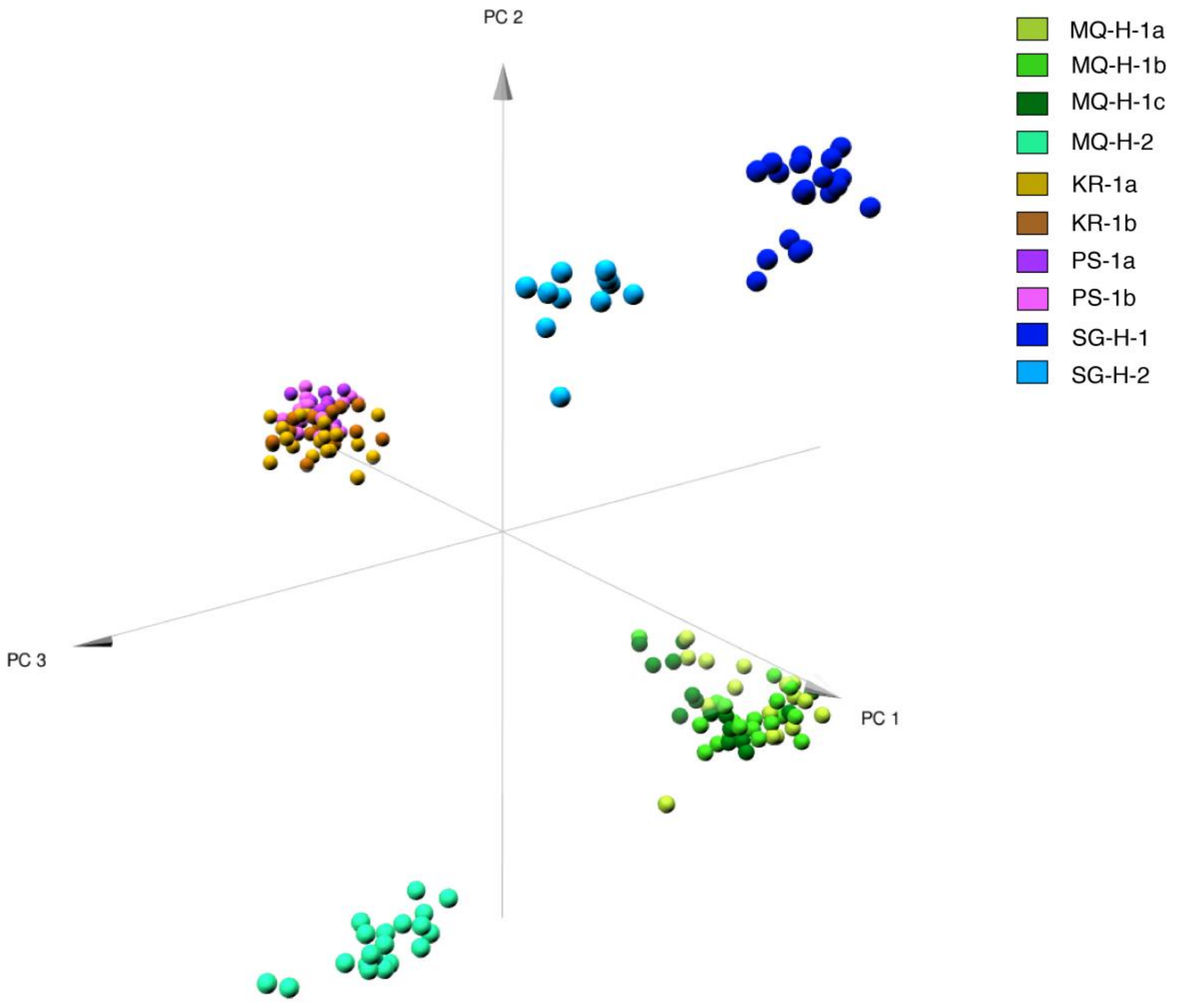

Figure S6 fastSTRUCTURE plot of *K*=3 identified clusters for *Hypogastrura viatica*, based on 7,275 SNPs. Each vertical bar shows the degree of membership of an individual to one of three clusters, represented by colours. The geographic source populations for individuals are indicated below the plot.

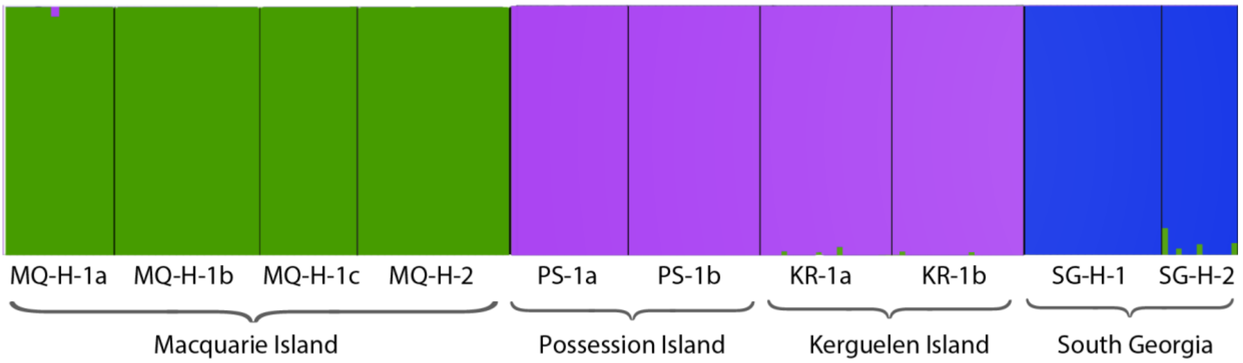

Supplement: Supplementary file 1 [file EVA-13-960-s001.pdf]
